# Learning to Estimate Dynamical State with Probabilistic Population Codes

**DOI:** 10.1371/journal.pcbi.1004554

**Published:** 2015-11-05

**Authors:** Joseph G. Makin, Benjamin K. Dichter, Philip N. Sabes

**Affiliations:** 1 Center for Integrative Neuroscience, University of California, San Francisco, San Francisco, California, United States of America; 2 Department of Physiology, University of California, San Francisco, San Francisco, California, United States of America; 3 UC Berkeley-UCSF Graduate Program in Bioengineering, University of California, San Francisco, San Francisco, California, United States of America; Duke University, UNITED STATES

## Abstract

Tracking moving objects, including one’s own body, is a fundamental ability of higher organisms, playing a central role in many perceptual and motor tasks. While it is unknown how the brain learns to follow and predict the dynamics of objects, it is known that this process of state estimation can be learned purely from the statistics of noisy observations. When the dynamics are simply linear with additive Gaussian noise, the optimal solution is the well known Kalman filter (KF), the parameters of which can be learned via latent-variable density estimation (the EM algorithm). The brain does not, however, directly manipulate matrices and vectors, but instead appears to represent probability distributions with the firing rates of population of neurons, “probabilistic population codes.” We show that a recurrent neural network—a modified form of an exponential family harmonium (EFH)—that takes a linear probabilistic population code as input can learn, without supervision, to estimate the state of a linear dynamical system. After observing a series of population responses (spike counts) to the position of a moving object, the network learns to represent the velocity of the object and forms nearly optimal predictions about the position at the next time-step. This result builds on our previous work showing that a similar network can learn to perform multisensory integration and coordinate transformations for static stimuli. The receptive fields of the trained network also make qualitative predictions about the developing and learning brain: tuning gradually emerges for higher-order dynamical states not explicitly present in the inputs, appearing as *delayed* tuning for the lower-order states.

## Author Summary

A basic task for animals is to track objects—predators, prey, even their own limbs—as they move through the world. Because the position estimates provided by the senses are not error-free, higher levels of performance can be, and are, achieved when the velocity and acceleration, as well as the position, of the object are taken into account. Likewise, tracking of limbs under voluntary control can be improved by considering the motor command that is (partially) responsible for its trajectory. Engineers have built tools to solve precisely these problems, and even to learn dynamical features of the object to be tracked. How does the brain do it? We show how artificial networks of neurons can learn to solve this task, simply by trying to become good predictive models of their incoming data—as long as some of those data are the activities of the neurons themselves at a fixed time delay, while the remainder (imperfectly) report the current position. The tracking scheme the network learns to use—keeping track of past positions; the corresponding receptive fields; and the manner in which they are learned, provide predictions for brain areas involved in tracking, like the posterior parietal cortex.

## Introduction

Over the last decade, neuroscience has come increasingly to believe that sensory systems represent not merely stimuli, but probability distributions over them. This conclusion follows from two observations. The first is that the apparent stochasticity of the response, **R**, of a population of neurons inherently represents the likelihood of the stimulus **s**: **R** ∼ *p*(**r**∣**s**) [1]. The second is that certain common computations essential to the function of many animals require keeping track of probability distributions over stimuli, rather than mere point estimates. For example, primates integrate information from multiple senses by weighting each sense by its reliability (inverse variance) [5, 6]. This framework has been used to hand-wire neural networks that integrate spatial information across sensory modalities and across time [2, 7, 8]. The more challenging problem faced by the brain, however, is to *learn* to perform these tasks.

We have recently shown [4, 9] that the problem of learning to integrate information about a common stimulus from multiple, unisensory populations of neurons can be solved by a neural network that implements a form of unsupervised learning called density estimation. Such a network learns to represent the joint probability density of the unisensory responses—to build a good model for these data—in terms of the activities of its downstream, multisensory units. For example [4], an exponential family harmonium (EFH) [3] trained on the activities of two populations of Gaussian-tuned, Poisson neurons (linear probabilistic population codes [2]) that tile their respective sensory spaces (visual and proprioceptive, e.g.) will learn to extract the “common cause” of these populations, encoding the stimulus in its hidden layer. In this case, the unisensory information available on a “trial” can be characterized by two means (best estimates) and two variances (inverse reliabilities); and the estimate extracted by the hidden units of the trained network is precisely the inverse-variance-weighted convex combination that primates appear in psychophysical studies to use.

Ecologically, however, the critical challenge is not typically to estimate the location of a static object, but to track the state of a dynamically changing environment. This task likewise requires reliability-weighted combination of information, in this case of the current sensory evidence and the current best estimate of the state given past information. But it is considerably more difficult, since its solution requires learning a predictive model of the dynamics, which is not explicitly encoded in the sensory reports. In the case of Gaussian noise and linear dynamics (LDS), this recursive process is described by the Kalman filter, the parameters of which can be acquired with well-known iterative learning schemes. How the brain learns to solve this problem, however, is unknown.

Here we propose a neural model that accomplishes this task. We show that by adding recurrent connections to an EFH similar to that used in [4], the network can learn to estimate the state of a dynamical system. For concreteness, we consider the problem of tracking the dynamical state of the upper limb, a necessary computation for accurate and precise movement planning and control. In this case, the neural circuit corresponds to the posterior parietal cortex (PPC), which appears to subserve state estimation [10, 11]; and its inputs are taken to be a population of proprioceptive neurons. The network’s performance can be quantified precisely by restricting our view to linear-Gaussian dynamics, where the filtering and learning problems have known optimal solutions (respectively, the Kalman filter and expectation-maximization, a maximum-likelihood algorithm). And indeed, performance approaches that optimum.

We then extend the network to controlled dynamical systems. Under the assumption that the controls are provided by motor cortex, these too are observed only noisily by PPC, in the form of efference copy, which the network must then learn to interpret as motor commands. State estimation is again close to optimal. In addition, the network is neurally plausible in both its representation of stimulus probabilities [2] and in the unsupervised learning procedure, which relies only on pairwise correlations between firing rates of connected neurons [12, 13]. Finally, the network makes two predictions about neural circuits that learn to perform state estimation: (1) During learning, position receptive fields will emerge before velocity receptive fields; or more generally, receptive fields will develop from lower- to higher-order states, especially when explicit information about the higher-order states is not in the inputs. (2) Filtering is implemented by tuning to past positions (or more generally, lower-order states), rather than tuning directly to velocity (or more generally, higher-order states).

## Results

### Network performance

#### The filtering problem

We present results for an uncontrolled and a controlled dynamical system. For both, the basic dynamical system is second-order (position, velocity), discrete-time, stochastic, and linear. The noise in state transitions is additive, white, and Gaussian. The “observation” at time *t* is the response ($R_{t}^{\theta }$) of a population of Poisson neurons, with Gaussian (bell-shaped) tuning curves that smoothly tile position. Since these neurons are taken to be reporting the proprioceptive sense, the “position” variable is the angle of a (single) joint, Θ. For the controlled system, there is a second population of Poisson neurons—carrying the “efference copy,” $R_{t}^{u}$—that smoothly tiles a space of input torques, **U**. Details appear in the methods section **Input-data generation**.

The task of tracking an object (*estimation*) is to provide, at time *t*, the best estimate of the location that can be computed from all the noisy observations from time 0 up to *t*. When current position depends on only a finite number *n* of past positions, this problem can be solved recursively: rather than retaining a history of all past observations, it is necessary to maintain only the current best estimate of the state (a vector whose dimension is set by *n*), and the reliabilities of these estimates (a covariance matrix). By artful design of the system (see Methods), we have arranged for the optimal estimate at time *t* to be computable in closed form (see below). We emphasize that this computation does *not* approximate the firing statistics of the Poisson observations as Gaussian; see the section **The optimal filtering distribution** for details.

The task of *learning* is to acquire the parameters that make it possible to carry out the estimation task. These parameters correspond to a dynamical system (e.g., the state transition matrix and the covariance of the state transition noise), and a model of how the states of this system give rise to observations. In our network, however, the parameters that are learned directly are the synaptic connections (weights) and the bias of each unit’s response function; the correspondence with the parameters of the dynamical system is not transparent (we explore it below).

#### The network

**Fig 1:**
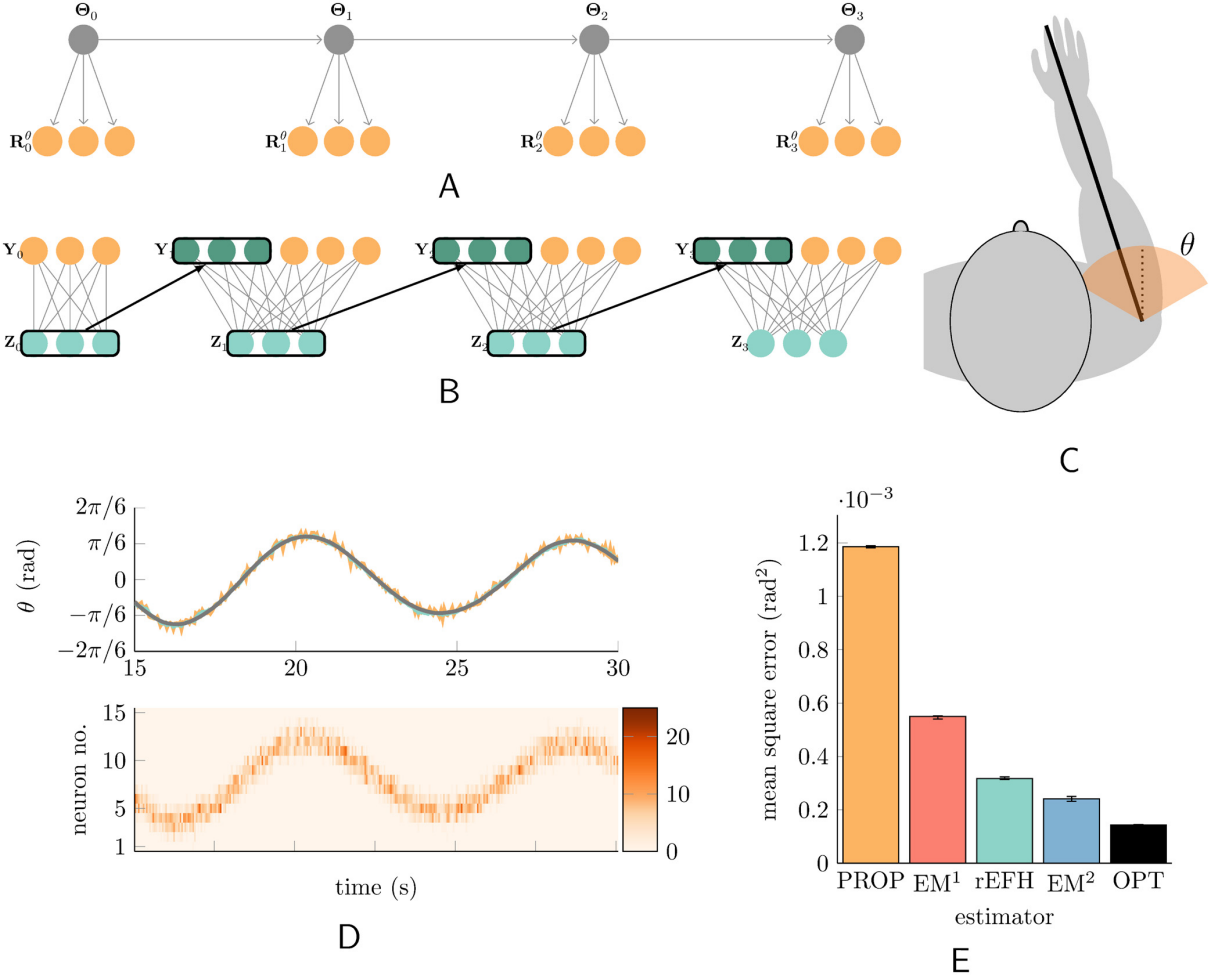
Dynamical system and the neural network that learns it. (A) The first four (of 1000) time steps of the linear system. The evolution is second-order, but only the current position (joint angle) is reported by the population of sensory neurons (orange)—fifteen of them, although only three are shown. (B) The input (“observed”) data for the harmonium (see text) at each time step are the current sensory activities, and recurrent activity of the hidden units, which amounts to a copy (heavy arrows) of their activity at the previous moment in time. (C) The joint angle. (D) (Top) Fifteen seconds of a typical trajectory (black) and the trajectory decoded from the position-encoding sensory population (orange), and from the hidden units (turquoise). (Bottom) The activity of the fifteen sensory neurons reported as a heat map. (E) Error statistics for angle decoding under different models (see text). Error bars mark the first and third quartile across twelve *de novo* trained and tested networks of each type (see Methods for training details).

The central idea behind training our network, the “recurrent, exponential-family harmonium” (rEFH), is the choice of input data. In particular, each input vector consists of both the proprioceptive response to the current joint angle, and the activities of the hidden units at the previous time step. (For the case of a controlled dynamical system, the input vector also contains a noisy copy of the efferent motor command.) Biologically, this could be implemented via an additional population that simply reports, at a single time-step delay, the activities of the hidden units (Fig 1B, heavy black arrows; see also the section **Cortical implementation** below). Conceptually, this choice of input data reflects the fact that filtering can be expressed as a “multisensory integration,” not between (e.g.) proprioceptive and visual inputs (cf. [4]), but between proprioceptive inputs and a running best estimate of the state. We hypothesize that, because the hidden units learn to extract all the information available in their inputs [9], the hidden vector at time *t* − 1 will accumulate the information of the filtering distribution, $p(\theta_{t-1}^{}|r_{0}^{\theta },\ldots ,r_{t-1}^{\theta })$. Then at the next time step, the network will “integrate” this information with the current proprioceptive information about joint angle. (See S2 Text for a longer discussion of this point.)

#### Experiments

We therefore test our network by decoding, for all *t*, hand position at time *t*, from its hidden units. Rather than directly compare this estimate to the optimal estimate, which would provide no sense of scale, we compute error statistics for both. That is, we take the difference between the network’s estimate and the *true* hand location; compute the mean and variance of this error across time steps (0 to *T* = 1000) and trajectories (*N*_traj_ = 40); and then compare these statistics for the rEFH and the optimum (OPT). We also compare error statistics from a “naïve” decoder (PROP) that simply decodes the current proprioceptive population, ignoring dynamics. It is the optimal decoder for data with no temporal dependencies.

The rEFH had to *learn* to solve the estimation problem, and in practice, learned solutions will always be somewhat suboptimal, because of finite, noisy data and a nonconvex problem space. Therefore, a perhaps more useful point of comparison is the set of the error statistics from another model that has been trained on the same data. In particular, it is possible in certain cases to derive optimal parameter-update equations for a learning procedure (“expectation-maximization,” EM) that is guaranteed to reach at least local optima. Again by design of the dynamical system, and although the rEFH is not in theory limited to such data, such update equations are available (they are derived in S1 Text). We therefore generate error statistics for this model (EM), as well. We emphasize, however, that EM was given additional information not provided to the rEFH: the order of the dynamical system, as well as the parameters of the observation model. The latter includes the best estimate of the stimulus given the population response, and the reliability of that “observation”—whereas the rEFH had to learn how to infer these values from the population response itself. Since EM is sensitive to the initial (random) values of the parameters it is to estimate, we present results for the best model from 20 random restarts (see Methods); the same was done for the rEFH. To determine what order of dynamics the rEFH has learned, we also compare against lower-order models trained with EM. The order of these models is denoted with a superscript (e.g., EM^2^).

#### Uncontrolled dynamical system

The generative model for the data appears in Fig 1A. Conceptually, the problem is to track the shoulder joint (Fig 1C). To encourage second-order behavior, the parameters of the system were chosen make it underdamped (as e.g. when the arm hangs downward and acts as a pendulum; see Methods), yielding trajectories like those shown in black in Fig 1D, and the proprioceptive responses shown in orange. The rEFH was trained as a density estimator on these responses and the recurrent activity of its own hidden units at the previous time step (Fig 1B).

Error statistics for the various decoders are shown in Fig 1E. Performance of the rEFH exceeds that of the naïve, purely sensory decoder (PROP), and approaches that of the optimum and the (second-order) EM-trained model (EM^2^). The rEFH also outperforms the first-order, EM-trained model, EM^1^, showing that it has learned to keep track not just of past positions, but of past velocities as well. We explore below *how* it encodes position and velocity (**Learned receptive fields and connectivity**).

**Fig 2:**
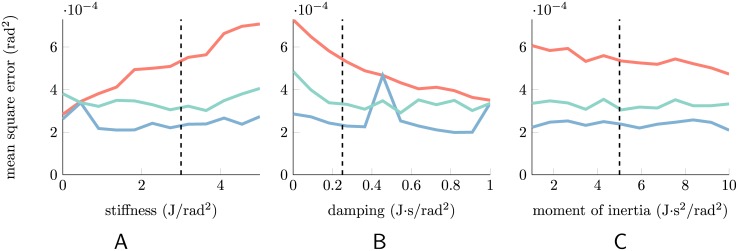
Mean squared errors (MSEs) for various dynamical systems. To show the flexibility of the rEFH, we train one for each of several different second-order dynamical systems. Each subplot shows the results for twelve different systems in which a single parameter has been varied across a range of values; otherwise the systems are identical to the undriven model of Fig 1. For each of those twelve dynamical systems, 20 rEFHs were trained (each with 150 hidden units), MSEs calculated, and the best selected (turquoise). The same was then done for EM^1^ (light red) and EM^2^ (blue), i.e., Kalman filters trained with EM, assuming either first- or second-order dynamics. The vertical black line in each plot indicates the dynamical system used in Fig 1 in the main text. (A) Varying the spring constant. The leftmost datum (*k* = 0) corresponds to the “no-spring” model from which the RFs are analyzed below (although with lower transition noise); at this point, the dynamics can be well approximated by a first-order model. (B) Varying the damping coefficient. The spike in EM^2^ at *c* = 0.4545 indicates that EM failed to find the second-order solution in any of its 20 attempts. As *c* increases and the systems approach critical damping, however, first-order approximations are increasingly adequate. (C) Varying the moment of inertia.

We chose the dynamics of the underlying stimulus because they let us clearly see that the rEFH can learn a second-order model; that is, it learns to track the lawful changes in velocity, as well as position—even though only position information was available at each time step. But the results are robust across various dynamical models. Fig 2 shows results for 36 different dynamical systems which were created by varying the oscillator’s Fig 2A stiffness, Fig 2B damping, or Fig 2C moment of inertia (colors as in Fig 1E). For all of them, the rEFH outperforms the first-order model (EM^1^), and performs close to the second-order model (EM^2^).

#### Controlled dynamical system

**Fig 3:**
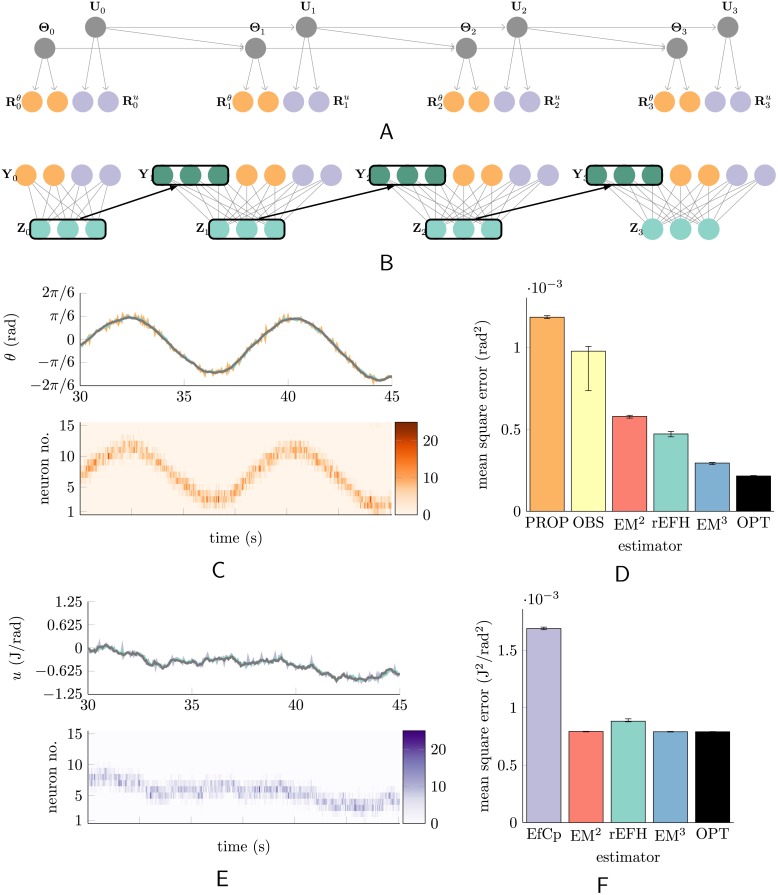
Dynamical system with efference copy. A reprise of Fig 1 for the controlled system. The evolution is now third-order, since the control signal is (close to) a random walk. (A) Angle (but not angular velocity) and the control signal are each noisily reported by populations of neurons (orange and purple; two of the fifteen neurons are depicted). (B) The training data for this harmonium at each time step are these two populations, and recurrent activity from the hidden units. (C) (Top) Fifteen seconds of a typical trajectory (black) and the trajectory decoded from the sensory population (orange), and from the hidden units (turquoise). The influence of the control can be seen in the (mildly) increasing amplitude of the oscillation. (Bottom) The activity of the fifteen sensory neurons is reported as a heat map. (D) Error statistics for joint-angle decoding under different models (see text). (E) The same as in (C), but the neurons carry “efference copy” rather than sensory information. (F) Error statistics for control-signal decoding under different models (see text). Error bars mark the first and third quartile across twelve *de novo* trained and tested networks of each type.

We now consider a system with inputs. Whereas the uncontrolled dynamical system, above, corresponds to the case where the arm is moved only passively by external forces, the controlled system corresponds to the more general case of self-motion. Controls are issued to the muscles of the arm by motor cortex, but a copy of these efferent signals is also fed back to posterior parietal cortex, Fig 3A [21]. This efference copy, being transmitted by a population of neurons, is assumed to be a noisy representation of the true control. Nor, presumably, is its role as a control signal explicitly given; rather, the network must learn, without supervision, to interpret it as such. This is precisely the learning problem faced by our network model (Fig 1B).

Realistic controls are correlated through time, so the control signal was given its own dynamics: a random walk with a very mild decay towards zero (see Methods). This resulted in trajectories like that in Fig 3E. The effect on angle can be seen in the corresponding trajectory of Fig 3C: here, the control is driving the trajectory increasingly negative (cf. the first and second trough) in spite of the damping and the restoring force. In general, since the control is a random walk rather than merely white noise, the changes it effects on the position trajectory tend to accumulate.

Mean squared errors (MSEs) for the various filters of the controlled system are shown in Fig 3D. The naïve model that ignores dynamics (PROP) is again the worst, as expected. Here the optimal EM-trained model is third-order (EM^3^), since the second-order dynamical system is driven by a control with first-order dynamics. Again the rEFH performs close to this model, and outperforms the best lower-order model (EM^2^). No trained model quite matches the true model’s performance (OPT), which result appears to be robust (see error bars), and presumably owes to the shape of the objective function (e.g., the optimal solution may be separated from suboptimal local maxima by deep valleys of low likelihood solutions).

So the rEFH has learned a third-order system. However, this does not *per se* show that it has learned to use the efference-copy population; it might, for example, simply attribute all input to the system as white noise. To demonstrate that it does learn to use the controls, we compare it to the *best* state estimates that can be made without efference copy. To produce such estimates, we fit a sixth filter, OBS, via regression, with full access to the state as well as the proprioceptive responses, but forced to assume zero control input. The resulting performance (Fig 3D, yellow bar) is clearly inferior to the other dynamical models.

Since the control signal is, like the state, only noisily observed by the network (via the efference copy), it is sensible to ask how well it can be decoded from the various models as well. And since the signal has its own (first-order) dynamics, it is possible for these models to make better estimates of the control at time *t* than can be made from the efference copy alone at *t*. Fig 3F shows that this is indeed the case. All the filters perform about equally well, in comparison with the non-dynamical decoding of the efference-copy population (“EfCp”), although the harmonium is slightly inferior.

#### Distributions of performance across initializations and hidden-layer size

One known limitation on the learning capacity of the harmonium is the number of hidden units: the network requires sufficient representational power in this vector to encode the cumulants of the input distribution—in this case, the filter distribution. To determine what network size is necessary for maximal performance, we test a series of networks with systematically increasing numbers of hidden units. Because, however, the number of recurrent units must equal the number of hidden units, the ratio of hidden units to input units (recurrent, proprioceptive, and efference-copy) is necessarily upper bounded at unity. This asymptote presumably diminishes the returns of additional hidden units, even beyond those limitations imposed by the learning algorithm or the difficulty of the filtering task.

**Fig 4:**
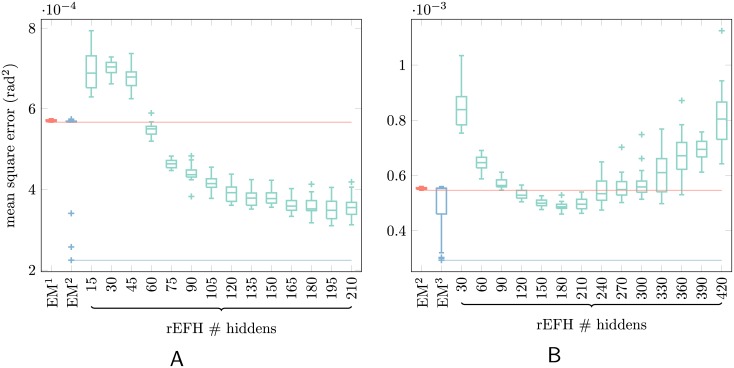
Box-and-whisker plot of MSEs for EM-based models and for rEFHs of various sizes. Each box corresponds to 20 networks trained *de novo* and tested on a common data set. Median MSE for each model is marked with a horizontal line; the box contains the interquartile range; whiskers extend to 1.5× the interquartile range, beyond which outliers are marked with plus signs. Performance among the EM-learned linear dynamical systems (the first two boxes, light red and blue) varies comparatively little, although large outliers are sometimes produced. In fact, the higher-order (best performing) models are all outliers. To facilitate comparison with the rEFHs, a line extends from the *best* EM-based models across the entire plot. The remaining twelve boxes (turquoise) are rEFHs with different numbers of hidden units, listed on the abscissae. All have the same number of recurrent units as hidden units, and have a fixed number of “sensory units”: (A) 15 proprioceptive, or (B) 15 proprioceptive and 15 efference-copy. (A) The uncontrolled dynamical system. Overall, MSEs decline with increasing number of hidden/recurrent units, but appear to asymptote by 180 hidden units (ratio = 180/15 = 12). (B) The controlled dynamical system. The optimal number of hidden units is about 180 (ratio = 180/30 = 6), after which mean and variance (across networks) of MSE increases.

For each of twelve sizes, we train 20 networks *de novo*, test them on a single fixed data set, and compute mean squared errors. The results are shown in Fig 4, where network sizes (abscissae) are given by the number of hidden units. For the uncontrolled network, Fig 4A, MSE of the best network (out of 20) clearly diminishes, asymptotically, with increasing numbers of hidden units. Beyond about 180 hidden units, no improvement in MSE is produced. For the controlled network, which has more to learn, the effect is even more severe (Fig 4B): increasing the number of hidden units beyond about 180 results in decreasing performance for even the best-performing networks at each network size. This suggests a limit to the complexity of the dynamical systems learned by this architecture, or with this learning procedure (for details of which, see Methods).

On the other hand, rEFH learning appears to be more robust than EM learning, as can be seen in the box plots for the EM-based models (Fig 4). As with the rEFHs, 20 of each of the four EM-based models were trained from scratch, resulting in the distributions shown in light red and blue in Fig 4A and 4B, (the narrowness of some of these distributions results in some very thin boxes). Although the EM algorithm guarantees convergence, it is only to a local (rather than global) optimum; which optimum is determined by the (random) initial parameters. The lower-order EM benchmarks (light red boxes; EM^1^ in Fig 4A and EM^2^ in Fig 4B) do indeed learn robustly, achieving nearly the same performance for all initializations. But the models with the true dynamical order (blue; EM^2^ in Fig 4A and EM^3^ in Fig 4B) exhibit a large performance distribution. These models are capable of outperforming the rEFH, but the runs that do are outliers. Thus, the large majority of the true-order EM-based models, for both the controlled and uncontrolled dynamical system, perform only about as well as their lower-order counterparts, which is inferior to all 20 of the 180-unit rEFH models. These rEFHs show comparatively little variation in performance—although that variance increases with the number of hidden units beyond 180.

### Learned receptive fields and connectivity

#### How does the rEFH track the state?

**Fig 5:**
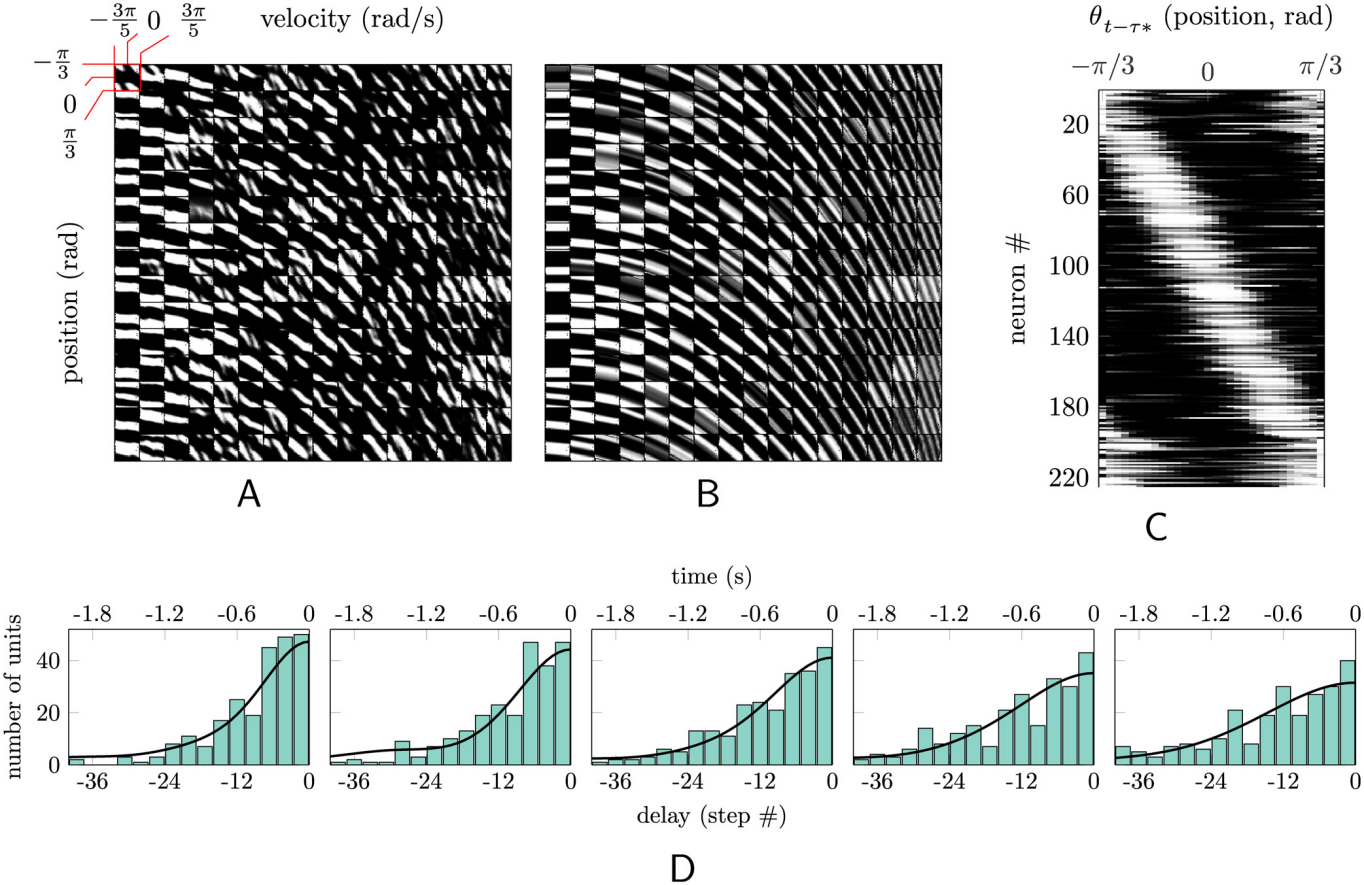
Position and velocity receptive fields of hidden units. In (A)-(C), pure white corresponds to a firing probability of one; pure black to zero. (A) Receptive fields for all 225 hidden units of the spring-free model (see text) in the space of (angular) position (ordinates) and velocity (abscissae). The angle limits and angular-velocity limits, indicated on the first (upper-left) receptive field, are the same for all units. (B) The predicted position-velocity receptive fields of units that have only the lagged-position tuning given by (C). The match with (A) is excellent for all but the anomalous 25 units at the right. (C) The same 225 units, each now plotted as a function of the time-lagged position with which that unit has maximal mutual information. Units have been arranged in order of increasing preferred position, whereas the units in (A) and (B) are arranged in order of maximally informative lags: from top to bottom and left to right, units are tuned for more temporally distant positions. This tuning gives rise to “stripes” in position-velocity space. For (A)-(C), the 25 units that do not appear to be well modeled by tuning to past positions have been placed at the end. (D) Histograms of the “preferred” lags, in terms both of time and (equivalently) discrete time steps, for five different networks. The normalized autocorrelation of the underlying dynamical system is superimposed. The central panel corresponds to the network analyzed in (A)-(C). The other four panels correspond to networks trained on observations from dynamical systems with different autocorrelations. From left to right panel, the dynamical systems get slower.

Optimal (or nearly optimal) position estimation for these dynamical systems requires tracking velocity and position, so we plot receptive fields (RFs) in position-velocity space. Now, for oscillatory dynamics, high speeds rarely co-occur with positions far from zero (equilibrium), which leaves the “corners” of such RFs empty. This obscures the pattern of RFs and the corresponding state-estimation scheme learned by the rEFH. Therefore, for simplicity, we present results from a network trained on a third dynamical model (“no-spring”): uncontrolled, and with no spring force (see Methods). (Similar results, albeit less clean, are observed in the corresponding analyses for oscillatory dynamics; see S3 Text in the supporting material.) In Fig 5A, the position-velocity receptive fields are plotted for all 225 hidden units of this rEFH, arranged in a 15 × 15 grid. The ordinate of each subsquare corresponds to position (increasing from top to bottom), and the abscissa to velocity (increasing from left to right). The large majority of receptive fields are negatively sloped “stripes” in this space. Interestingly, they resemble in this the receptive fields of neurons in MSTd of a rhesus macaque trained to track moving stimuli [22]—although in that work there are positively-sloped stripes as well.

Interpretation of these receptive fields is facilitated by an observation. If the velocity (*ω*_*t*_) is roughly constant over *n* time steps of length Δ, then:

$$\begin{array}{cc} \omega_{t-1} & \approx \frac{\theta_{t}^{}-\theta_{t-n\Delta }^{}}{n\Delta }\\ \Rightarrow \theta_{t}^{} & \approx n\Delta \omega_{t-1}+\theta_{t-n\Delta }^{}, \end{array}$$

the equation of a line in position-velocity space. Hence, such fields could be produced by neurons tuned simply for position at a delay, where the size of the delay (*n*Δ) determines the slope (negative because we have plotted position as increasing from top to bottom, to match the corresponding figure in [22]), and the “preferred” position determines the y-intercept. The equation is exact for *n* = 1; but to the extent that velocity is not constant, the receptive fields will be diminished—as seen in the more irregular and faded character of receptive fields with greater slopes.

We therefore re-plot the receptive fields as a function of position only, but each at the time delay that maximizes mutual information between position and that hidden unit’s response (Fig 5C). Units are ordered by preferred position (whereas units in Fig 5A were ordered by time delay). The resulting position tuning curves appear to tile space uniformly, with roughly constant receptive-field widths, suggesting that this is a concise description of the tuning curves that captures the computation being performed. As a final method of verification, we use these lagged-position receptive fields to generate idealized position-velocity receptive fields (Fig 5B; see **Tuning analysis** for details.) The match with Fig 5A is apparent.

This result is, perhaps, unexpected. It is possible to represent (an estimate of) the current state of a second-order dynamical system compactly by encoding current position and the current velocity. But it is also possible to represent it—seemingly less efficiently—in terms of the past positions alone, with the weight on each position decaying exponentially as a function of the number of time steps into the past. The rEFH appears to have learned a representation of this second type.

To determine if the weighting function applied to past positions by the rEFH does indeed correspond to such a scheme, we examine the distribution of “preferred” lags across hidden units (Fig 5D, center panel). Unsurprisingly, most units are tuned to the recent past, with an apparently monotonic decline into the past. Superimposed is the autocorrelation of the dynamical system on which the network was trained (see Methods), normalized to have the same integral as the histogram. Evidently, the distribution of lags is well tuned to the dynamics. To confirm the robustness of this finding, we trained four new rEFHs on four different dynamical systems, identical to the one under discussion up to the damping coefficient (frictional force). From left to right in Fig 5D, the dynamical systems were increasingly damped, resulting in longer autocorrelations (thick black lines). The temporal tuning of the rEFHs trained on these dynamical systems appears well matched in each case.

#### Responsiveness to instantaneous reliability

Thus, the rEFH tracks stimuli by encoding its position at various lags, with the number of units assigned to each lag decreasing exponentially with distance into the past, according to the autocorrelation of the signal. What is nevertheless not clear from Fig 5D is whether the rEFH’s weighting of past positions takes into account the *instantaneous* reliability of the proprioceptive encoding of joint angle. In our models, as (presumably) in the brain, instantaneous reliability varies because the proprioceptive report of joint angle is corrupted by Poisson noise. For Poisson spiking, the reliability—i.e., the inverse variance of the posterior distribution over joint location, conditioned on the spiking of all proprioceptive neurons—is proportional to the total number of spikes produced by the population at that time step. In the results discussed above, we also increased the fluctuations in this number by additionally varying the “gain” of the proprioceptive population, i.e., the single parameter that sets the height of all the tuning curves (see **Input-data generation**). This is meant to model other random changes in the reliability of the proprioceptive report. Thus the optimal weighting of past position information, although on average an exponentially decaying function of time delay, will at any particular moment vary as a function of the recent, unpredictable, history of reliabilities: higher weights should be assigned to those time steps when the proprioceptive units had a collectively higher average firing rate, and *vice versa*. A network that ignores such fluctuations will perform well, but suboptimally, perhaps explaining the (small) discrepancy between the MSEs for the rEFH and EM^2^ seen in Fig 1E.

**Fig 6:**
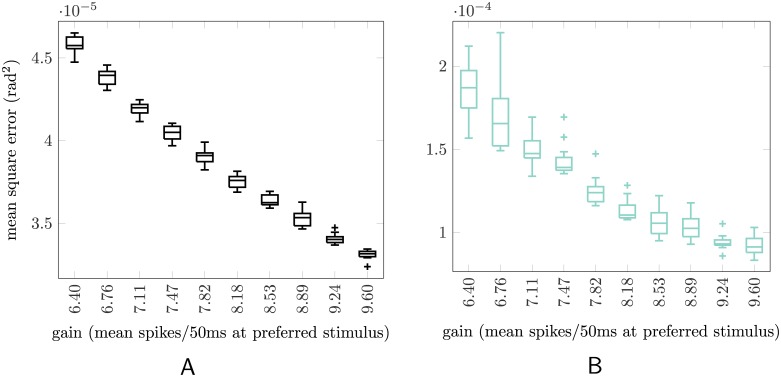
Network sensitivity to instantaneous reliability. The instantaneous (one-time-step) reliability of sensory information is determined by the total number of spikes across the sensory population within one time step. An optimal filter will up-weight sensory information that is more reliable (and vice versa). If such a filter is run on *noiseless* sensory data, then its errors will be smaller for sensory input with more total spikes (higher gain), since it will up-weight the perfect sensory information. (A) Box-and-whisker plot (interpretation as in Fig 4) of mean squared errors for the optimal model (OPT), when tested on noiseless sensory data and a range of gains. For each gain on the abscissa, the filter was tested on 12 sets of 320 trajectories apiece, for which the sensory gain was fixed throughout. Higher-gain trajectories yield lower mean errors, as expected. (B) The same plot for the network (rEFH). The magnitude of the MSEs is larger than for the optimal filter, as in Fig 1E, but the pattern is the same, showing that the rEFH has indeed learned to treat higher-gain (more-spikes) sensory information as more reliable.

Here we show that the rEFH is indeed sensitive to instantaneous reliability. We retest the network of the section **Uncontrolled dynamical system** on *noiseless* sensory data, i.e., using the mean spike counts of the proprioceptive neurons rather than Poisson samples drawn from those means. This allows us to set the total spike count essentially directly. In this noiseless test, a higher total spike count does not correspond to a more reliable signal: the signal is perfectly reliable for all spike counts. Hence for any total spike count, minimal (zero) error could be achieved by a “filter” that relied entirely on the current sensory input. Nevertheless, the optimal filter *for the data on which the rEFH was trained*, viz., OPT, will *not* rely entirely upon this current sensory information, but rather will weight it in proportion to the total spike count. In consequence, OPT will achieve lower MSEs on data with greater total spike counts, since for such data it will lean more heavily on the perfect sensory information. This is the pattern observed in Fig 6A. If the rEFH is also treating total spike count correctly—i.e., if it is properly sensitive to instantaneous reliability—its MSEs will exhibit a similar pattern. And indeed, they do (Fig 6B).

Here we emphasize again that, for the optimal filter (as well as OBS and EM^*n*^), we provided the transformation from total spike count to sensory reliability, whereas the the rEFH had to learn this transformation. Likewise, in engineered solutions to tracking problems, the Kalman filter is usually simplified to learn a single, average reliability for all time. We have demonstrated that the rEFH is not similarly limited, since it can learn to use instantaneous indicators of reliability if they are present in the observations.

#### Emergence of receptive fields

**Fig 7:**
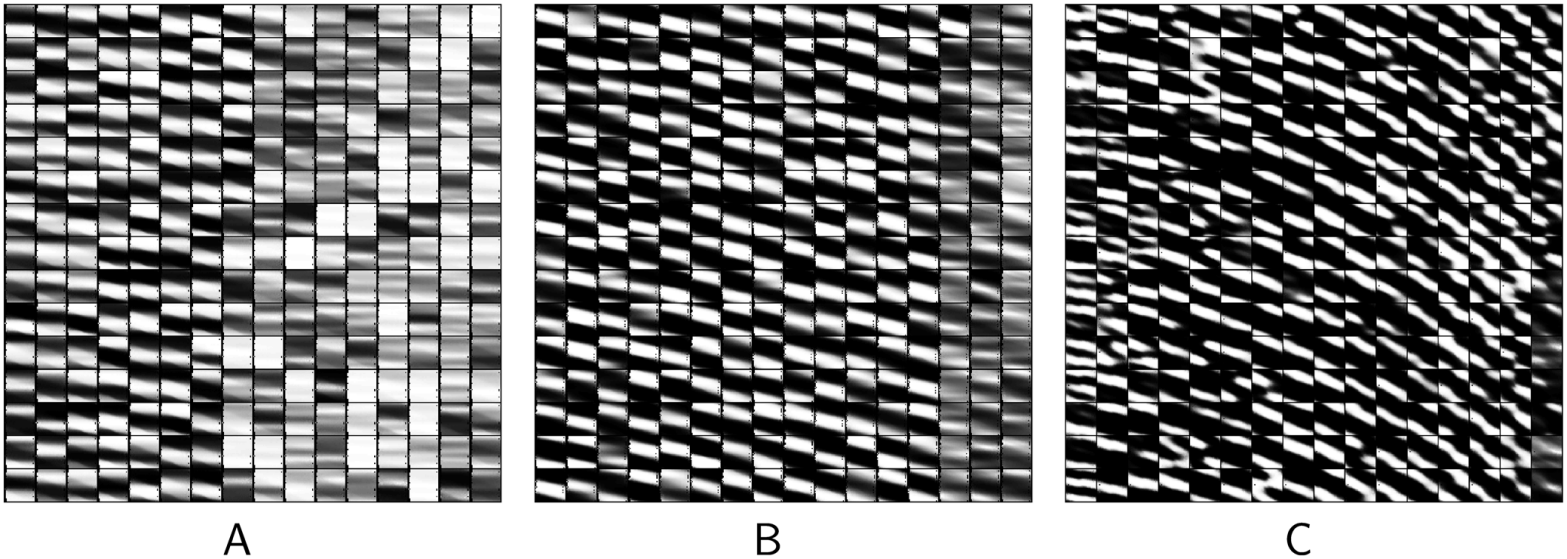
Emergence of position and velocity receptive during training. Velocity tuning takes longer to emerge than position tuning, because velocity information is only available in the inputs after position has already been learned by the hidden units—and subsequently fed back. (A) After 100 batches of training, stripes are mostly horizontal or very shallowly sloped in position-velocity space—no velocity tuning. (B) By 200 batches, velocity tuning is evident across most units. (C) Later, at 1000 batches, the slopes of the “stripes” have increased, indicating position tuning for more temporally distant (past) stimuli.

Since velocity is not reported directly by the sensory (“proprioceptive”) population, the network will not immediately develop tuning for it. Fig 7 illustrates its emergence across training trials. (We return here to the “no-spring” model for comparison with Fig 5A.) Since position information is a useful “feature” for explaining the proprioceptive inputs, the hidden units learn to extract it after just 100 batches of training (Fig 7A). But information that is in the hidden units will also appear in the input, at a one step time delay, via the recurrent units (see Fig 1B). So at this point in training, the input contains information about both the current position (in the proprioceptive population) and about the past position (in the recurrent population). This makes extraction of velocity information possible. It is *useful* because the stimuli obey second-order dynamics: knowing the relationship between past and present position allows each to provide information about the other, yielding overall superior estimates. And indeed, by 200 batches of learning, some velocity tuning appears, evidenced by the sloping of receptive fields in position-velocity space, Fig 7B. But these units look back no more than a few steps in time. By 1000 batches (Fig 7C), strong velocity tuning is evident, although the full distribution of lags (slopes) has yet to emerge (cf. Fig 5A, which is after some 100,000 batches of training).

#### Organization of the learned weight matrix

**Fig 8:**
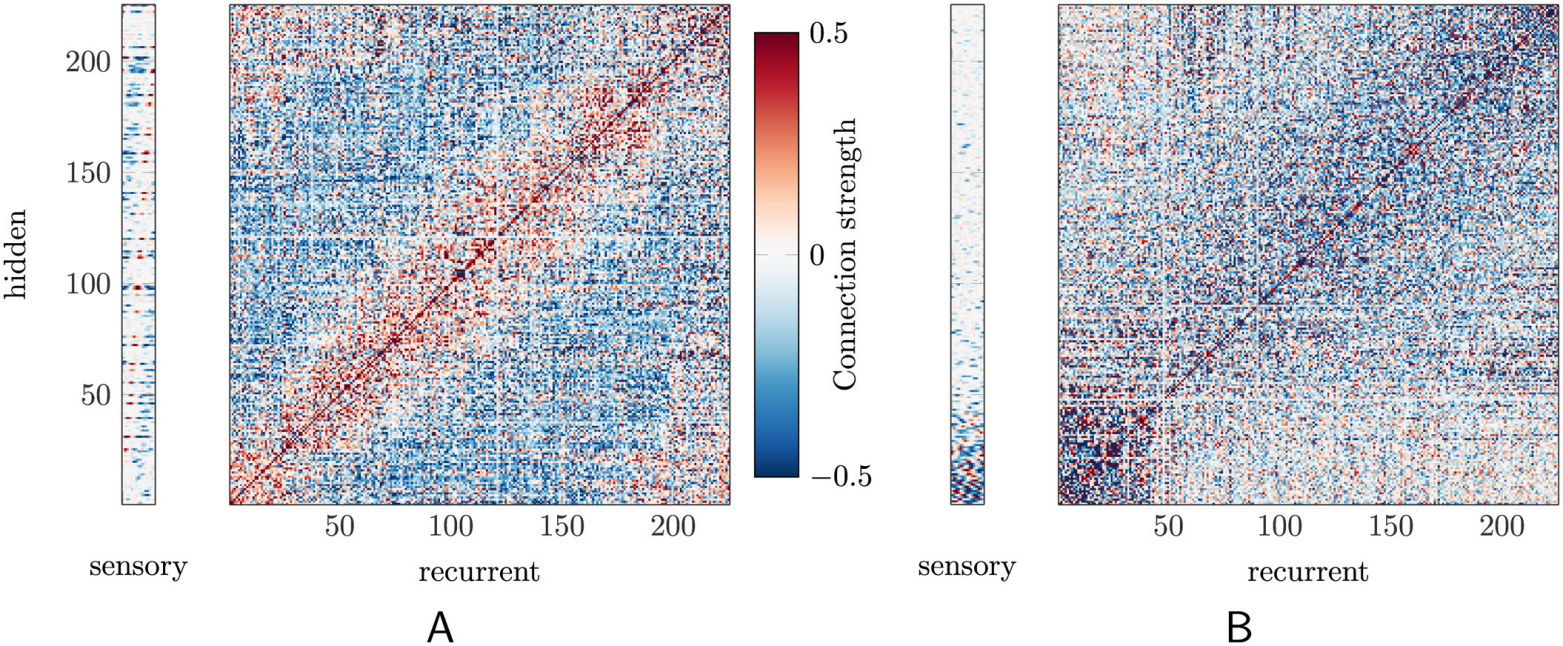
The weight matrices. The organization of connections between inputs (sensory or recurrent units) and the hidden layer can be visualized by sorting units by (A) preferred stimulus angle or (B) by preferred lag (increasing from lower left to upper right). Here we analyze the “no-spring” network (see text). In both subfigures, both the recurrent and hidden units have been re-sorted; the sensory units remain organized by preferred angle. Note that self-connections (along the diagonal) are in fact more than 0.5, but the plot saturates at this value to make the other connections more visible.

The network learns to model the dynamics by making changes to the synaptic connection strengths, summarized by the weight matrices *W*_prop_ (sensory to hidden) and *W*_fb_ (recurrent to hidden). To understand better the mechanism of the network we examine these matrices (Fig 8), again for the “no-spring” model. (The corresponding figure for the non-zero stiffness model, slightly more difficult to interpret, is shown in S3 Text.) In the arbitrarily ordered form in which they are learned, they are difficult to decipher, but interesting patterns emerge when they are reordered by the parameters of the receptive fields. The reordering is applied to both the hidden and the recurrent units. (The original, topographical ordering of the sensory units is retained.)

First, reordering by the hidden units’ preferred stimulus angles (“PA”), Fig 8A, reveals that the difference in PA between two hidden units dictates the sign of their connection. Hidden units with similar PAs have positive connections, and units with “out of phase” (recall that the stimulus is a circular variable) PAs have negative connections. This results from the continuity of the stimulus trajectories: the network “expects” the stimulus to move from any given position (encoded in the recurrent units) to a nearby one (encoded in the hidden units).

Second, we reorder the units by “preferred lag,” *τ*—i.e., the time delay at which mutual information between sensory input and hidden-unit activity is maximal (Fig 8B). Again, units with similar *τ* are preferentially wired together.

### Cortical implementation

More than one cortical area is thought to subserve object tracking. Since we have in this study focused on the task of tracking one’s own limbs, we consider posterior parietal cortex (PPC), which is thought to be responsible for this task [10, 11]. The computation may well be distributed across the PPC, but we focus on just one that has been particularly implicated [11], Brodmann Area 5. Our aim is to show that our neural network and its learning scheme are consistent with what is known about the connectivity of Area 5, both interlaminar and inter-areal. In particular, we consider its connections with the primary motor area (M1) and primary somatosensory cortex (S1). Our proposed implementation is speculative and not the only one possible; e.g., we identify the “recurrent” units with another layer of Area 5, but they might alternatively correspond to another area of PPC.

**Fig 9:**
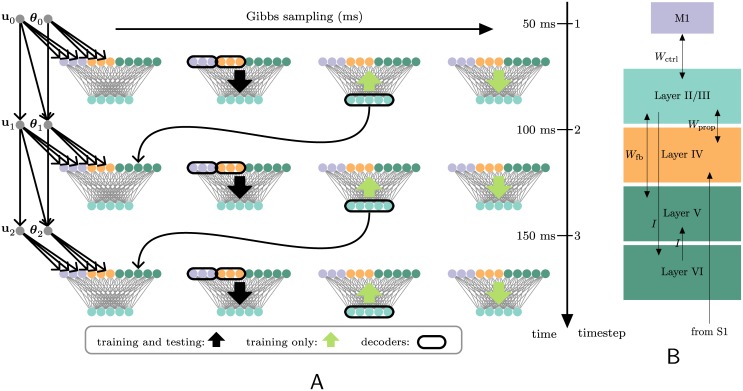
Training and testing: in the model, and in a cortical implementation. (A) The training and testing procedure in the model. Three discrete times steps are arrayed vertically. At each one, the current arm position (**θ**_*t*_) is reported by the proprioceptive population (orange) with Poisson-distributed spike counts, as shown in the first column. The current motor command is likewise reported by an “efference-copy” population (purple). Second column: this spiking, along with recurrent activities (dark turqoise), stochastically drives single spikes in the hidden layer (turquoise). Third column: these spikes in turn drive the three “input” populations (this is not required during testing); a “copy” of the hidden vector is also saved to serve as recurrent activity at the next time step (curved black arrow). Fourth column: finally, the input populations drive the hidden layer once more, after which the weights are changed according to Eq. 9 (also not required during testing). At every time step, the current joint angle and current control are decoded naïvely from the current activities of their respective input populations; with Kalman filters that are recursively updated based on these activities (not depicted in this figure); and from the hidden units of the rEFH. (B) A possible cortical implementation of the rEFH. The cortical layers of Brodmann Area 5 and its inputs are identified with elements of the rEFH by color. The synaptic connections are denoted by the arrows and their corresponding weight matrices. Primary somatosensory cortex (S1) provides feedforward proprioceptive input to layer IV, while primary motor cortex (M1) provides feedback input—a copy of the efferent command—to layer II/III. The recurrent signal of the rEFH (heavy curved arrow in (A)) is identified with a reverberatory loop from II/III to VI, to V, and back to II/III.

Fig 9A summarizes the training procedure from an algorithmic perspective (see Methods for details). In Fig 9B, as in Fig 9A, input comes from two sources. Feedforward, proprioceptive input ($R_{t}^{\theta }$) from primary somatosensory cortex, S1 (especially BA3a), projects to layer IV [23]. A copy of the efferent command ($R_{t}^{u}$) feeds back from M1 to layer I of Area 5 [23]. Layer II/III of Area 5 in turn projects forward to M1 [24]. Layer I is not believed to contain cell bodies [25], so we take these to be the terminal branches of the apical dendrites of layer II/III cells (which are also lightly labeled by anterograde tracers injected in M1 [23]). Within Area 5, we propose that the temporally delayed recurrency (**Z**_*t*−1_) of the rEFH is provided by the loop from layer II/III down to VI, then up to V, before modulating the activity of layer II/III neurons, consistent with the anatomy of Area 5 [25]. Layer IV and III, as well as V and III, also have reciprocal connections [25], as required for the rEFH training procedure. The latter loop has in fact been hypothesized to give rise to rhythmic activity in rat parietal cortex [26].

According to the learning and filtering schemes of our model, the temporal flow of information is as follows. Sensory input ($r_{t}^{\theta }$) and efference copy ($r_{t}^{u}$) arrive at, respectively, layer IV of BA5 and the feedback layer (presumably VI) of M1. At the same time, a “copy” (which could be any information-preserving transformation) of activity from layer II/III of BA5 (**z**_*t*-1_) passes down to layer V. Next, the spiking in these layers (M1 layer VI, BA5 layer IV, BA5 layer V) drives spiking (**z**_*t*_) in BA5 layer II/III. These responses encode, according to the model, the optimal estimate of the limb, and this information will ultimately become the temporally delayed recurrent activities identified above. For learning, however, it is also necessary that this activity drive spiking in M1 (${\hat{r}}_{t}^{u}$), BA5 layer IV (${\hat{r}}_{t}^{\theta }$), and BA5 layer V (${\hat{z_{}^{}}}_{t}$), through the reciprocal connectivity lately noted. A “copy” of the layer II/III activity (**z**_*t*_) is simultaneously propagated down to layer VI. Lastly, the activities in M1, BA5 layer IV, and BA5 layer V again drive activity (${\hat{z_{}^{}}}_{t}$) in BA5 layer II/III. At the same time, the “copy” of layer II/III activity (**z**_*t*_) is communicated up to layer V.

## Discussion

### Summary of results

We have shown that a neural network (the “rEFH”) with a biologically plausible architecture and synaptic-plasticity rule can learn to track moving stimuli, in the sense that its downstream (“hidden”) units learn to encode (nearly) the most accurate estimate of stimulus location possible, given the entire history of incoming sensory information (Figs 1 and 2). This requires learning a model of the stimulus dynamics. This is (as far as we know) the first biologically plausible model that has been shown to learn to solve this task. Moreover, the network learns the *reliability* of the sensory signal: the trained network leans more heavily on the internal model when the sensory signal is less reliable, and more heavily on the sensory signal when it is more reliable (Fig 6).

We are particularly interested in tracking the state of one’s own limbs. Here, additional information about stimulus location is thought to be available in the form of a “copy,” relayed to the posterior parietal cortex, of the efferent motor command [21]. And indeed, when such signals are available to our network, it learns to make use of them appropriately to track the arm more precisely—in spite of the fact that none of the incoming signals is “labeled” according to its role (Fig 3). Although an expectation-maximization (EM) algorithm can sometimes learn a Kalman filter that noticeably outperforms the best rEFH on these data, it usually does not (Fig 4). That is, learning in the rEFH is more robust than EM in the sense that the variance in performance across models trained *de novo* is smaller, albeit at the price of a bias towards worse models. Finally, and surprisingly, the downstream neurons of the trained network track a moving stimulus by encoding its position at various time lags (Fig 5).

### Related work

The earliest implementation of dynamical state estimation (“filtering”) in neural architecture comes from Rao and Ballard [27]. Their model, like ours, assigns a central role to recurrent connections, but as predictive coders rather than simply delayed copies of previous neural states. Likewise, the network connectivity is acquired with an unsupervised and local learning rule, a variant on EM. However, the authors do not train their network on moving objects or moving images, presumably because convergence of the neural state under their learning scheme is slow compared with any plausible stimulus dynamics. Instead, the connectivity is acquired on static images. Performance on state-estimation tasks is not tested.

Several groups have hand wired neural networks to act as state estimators [7, 8, 28]. Although these papers do not address our central concern, the learning problem, it is nevertheless useful to compare the resulting architectures with our rEFH. For example, Beck and colleagues constructed a neural network to implement the Kalman filter on linear probabilistic population codes, as in this work, and showed its performance (measured in information loss) to be nearly optimal. From analytical considerations, the authors showed that the required operations on neural firing rates are weighted summation (as in our network) and a quadratic operation (that acts like a divisive normalization in the steady state). In our rEFH, on the other hand, the only nonlinearities are elementwise: interaction between inputs is always in the form of a weighted sum. That the rEFH can nevertheless filter (nearly) optimally is possible because we do not require, as they do, that the output population encode information in the same way as the inputs (sc., that the posterior distribution over the stimulus have linear sufficient statistics; see S4 Text). This critical difference provides the basis for an experimental discrimination between the respective models. Likewise, filters have been hand wired into attractor networks [28] and spike-based (rather than rate-based) networks [8]. The latter in particular argues that the precise arrival time of spikes contains information about the stimulus, rather than the average rate across time, as in in our model.

An approach that does include learning comes from Huys, Zemel, Natarajan, and Dayan [29, 30]. The authors formulate the problem in terms very similar to ours, but they allow more general dynamical systems generated by Gaussian processes, and the basic unit of information is spikes rather than spike counts (although approximations that ignore precise arrival times lose little information [29]). The most significant difference with our work is that the authors learn the parameters of their network with a supervised, non-local rule, which they do not consider to be a biological mechanism. But again the comparison is instructive. We are able to formulate an unsupervised rule because we approach the filtering problem indirectly: Natarajan and colleagues require the posterior distribution, conditioned on hidden-unit activities, to be factorizable over hidden-unit spikes (so that a third layer can consider those spikes separately), and then force it to match the true filtering distribution by directly descending the KL divergence between them [30]. We, on the other hand, force the network to be a good model of its incoming data—which, when some of those data are past hidden-unit activities, achieves the same end.

In the machine-learning literature, Hinton and colleagues have proposed three variants on a theme quite similar to ours [31–33], although different in important ways. Most importantly, in all three, the past hidden-unit activities are treated by the learning rule as (fixed) biases rather than as input data; i.e., they cannot be modified during the “down pass” of contrastive-divergence training. That these activities ought to be treated as data, we argue more rigorously in a forthcoming work.

The earliest variant [31], the “spiking Boltzmann machine,” is, like ours, a temporal extension of the restricted Boltzmann machine that is trained with the contrastive-divergence rule. Hidden units are directly influenced by past hidden-unit activities, as with the rEFH, but possibly from temporal distances *τ* that are greater than one time step (*contra* the rEFH). However, the weights from a particular “past” hidden unit at various delays (e.g., from $z_{t-n\tau }^{i},n\in \left\{1,2,...\right\}$ to $z_{t}^{j}$) are constrained to be identical up to a fixed (not learned) exponential decay. The motivation was to model the influence of past spikes in a biologically plausible way: Whereas in our rEFH, the (one-time-step delayed) past hidden activities are maintained in a separate population of neurons (Fig 9), in the “spiking Boltzmann machine” their effect on current hidden units is interpreted simply as the decaying influence of their original arrival. This makes it plausible, unlike in the rEFH, to include influences at delays greater than one time step. On the other hand, it necessitates treating those effects as biases rather than data.

It is difficult to judge the limitations this imposes on the model, since the authors do not quantify its performance. However, they do investigate more thoroughly performance of a similar, but more powerful network. The “temporal restricted Boltzmann machine” (TRBM) [32] is a spiking Boltzmann machine without the constraint that the weights decay exponentially backwards in time; instead, they are learned freely and independently for all time. The order of the dynamical system that can be learned by this network turns out, unlike ours, to be tied to *τ*: TRBMs with *τ* = 1 (like the rEFH) can learn only random-walk behavior (first-order dynamics) [33]. This can (presumably) be overcome by including connections back as many time steps as the order of the system to be learned, but it is not obvious what biological mechanism could maintain copies of past activities at distant lags, or determine *a priori* how many such lags to maintain. The same authors show that this problem can be alleviated with a variant architecture, the “recurrent temporal RBM” (RTRBM) [33], but it requires a non-causal learning rule (backpropagation through time), again making it a poor model for neural function. For neither model do the authors precisely quantify its filtering performance; we do in a forthcoming study.

### Implications of the model

Our simulations demonstrate three things: First, the rEFH is capable of learning to “track” moving stimuli, i.e. to estimate their dynamical state, and nearly as well as an optimal algorithm, as has been seen behaviorally in humans [34]. In fact, the network learns to encode the full posterior distribution over the stimulus, rather than just its peak: although we did not show it directly, it must, since the variance of this (Gaussian) distribution is required to combine properly the previous best estimate with the current sensory information. And rather than relying on a fixed estimate of sensory reliability, the network learns to take into account instantaneous changes in it (Fig 6).

Second, the network does not require a special architecture or *ad hoc* modifications. It is, rather, identical, up to the choice of input populations, to the network and learning rule in our previous work [4]. Thus, if the input populations are proprioceptive and *recurrent* units, it will learn to estimate dynamical state; if they also include efference copy, it will learn the influence of motor commands on stimulus dynamics. If they are proprioceptive and *visual* reports of a common stimulus, it will learn to perform multisensory integration; if a gaze-angle-reporting population is also present, to transform the visual signal by that angle before integrating (“coordinate transformations”); if the stimulus distribution is non-uniform, to encode that distribution [4]. (We have shown elsewhere, in terms of information theory, why this is the case [9]. For further discussion of the relationship between the static and dynamical computations, see S2 Text.) Thus, the network provides a very general model for posterior parietal cortex, where some combination of all of these signals is often present.

Third, the model makes some predictions about the encoding scheme, receptive fields, and connectivity of cortical areas that track objects. As with all models, we take certain elements of ours to be essential and others to be adventitious. That learning in posterior-parietal circuits can be well described as a form of latent-variable density estimation, for example, is central to our theory; but the precise form of the learning rule (“one-step contrastive divergence”), although plausible, is not. Our theory requires that sensory neurons encode distributions over stimulus position, but the representation scheme need not be probabilistic population codes of the Pougetian variety [2]. Here we list three predictions that do follow from essential aspects of the network.

1. The network learns to track by encoding past positions. This is a non-obvious scheme (it is not, e.g., the one used by the Kalman filter) and apparently results from the fact that only position information is reported by the sensory afferents. It is possible that such receptive fields (Fig 5A) are in fact found in MSTd of monkeys that have been trained to track moving objects [22]. Now, in many circuits, velocity is detected at early stages. But even when velocity is directly reported by the inputs to an rEFH, tuning to past positions still appears, albeit with lower prevalence (see S3 Text).
  More generally, we predict that higher derivatives (e.g., acceleration), especially those not directly available in sensory input, will be encoded via delayed, lower derivatives (e.g., velocities)—as long as those higher-order states have lawful dynamics.
2. During learning, receptive fields for position emerge before those for velocity. This is a necessary consequence of density estimation on recurrent units. A similar proviso attaches: where velocity information is directly reported, it is acceleration-coding that will emerge over time.
3. The use of delayed, feedback connections in neural circuits is a mechanism for learning dynamical properties of stimuli. Under this prediction, primary sensory areas that process information with very little temporal structure—e.g., smell—will lack the dense feedback found in, e.g., visual areas. Alternatively, the recurrency might be identified with interlaminar, rather than interareal, structure, as we have hypothesized (Fig 9B)—which would explain why piriform cortex only needs three layers.

### Neural computation in posterior parietal cortex

More generally, our investigation was motivated by two main ideas. The first is that populations of neurons, in virtue of their natural variability, encode probability distributions over stimuli (rather than point estimates) [1, 2]. Encoding certainty or “reliability” is a necessity for optimal integration of dynamic sensory information, since it determines the relative weight given to (a) current sensory information and (b) the prediction of the internal model. But rather than *explicitly* encoding the reliability of the stimulus location—e.g., via neurons that are “tuned” to reliability, as other neurons are tuned to location itself—this reliability is identified with the inverse variance of the posterior distribution over the stimulus, *p*(**s**∣**r**), conditioned on the population activity [2]. This distribution arises as a natural consequence of the (putative) fact that neural responses are noisy, and can therefore be characterized by a likelihood, *p*(**r**∣**s**) [1]. If reliability were not encoded this way, our learning scheme would not work: it would have no way of knowing what to do with those reliabilities, which would be to it indistinguishable from (e.g.) the location of another stimulus.

The second idea is that higher sensory areas, like posterior parietal cortex and MSTd, can encode more precise distributions over the location (e.g.) of a stimulus than that provided by their sensory afferents at any given moment in time. This is due, essentially, to the continuity of the physical world: at successive moments in time, objects tend to remain near their previous positions. More precise localizations can consequently be achieved by a form of averaging that, because objects do move, accounts for the predictable changes in position from moment to moment. This requires learning a model of those predictable changes. The rich statistical structure of the sensory afferents—including efference copy of motor commands that may be influencing the evolution of the stimulus to be tracked, as when tracking one’s own limbs—makes it possible to learn the model from those inputs alone. This unsupervised learning is a much more efficient approach than trying to use the few bits of information that may be available in the form of reward: very few rewards can be reaped before an animal can control its own limbs.

In the special case of linear dynamics and Gaussian noise, these two problems—learning a dynamical model, and filtering in that model—have known algorithmic solutions: an expectation-maximization algorithm and the Kalman filter, respectively. Rather than try to map operations on vectors and matrices directly onto neural activity and learning rules, we have taken a more general approach, showing how a rather general neural-network architecture that tries to build good models for its inputs can learn to solve the problem, if those inputs are suitably chosen: temporally delayed recurrent activity from downstream units must be among the inputs. Our network learns by a local, Hebbian rule operating on spike-count correlations, although it remains to relate these to more specific biological learning rules, like STDP.

## Methods

Notation is standard: capital letters for random variables, lowercase for their realizations; boldfaced font for vectors, italic for scalars. Capitalized italics are also used for matrices (context distinguishes them from random scalars).

### Input-data generation

We describe the most general dynamical system and observation model to be learned: a controlled, second-order, discrete-time, stochastic, linear dynamical system, whose “observations” or outputs come in the form of linear probabilistic population codes [2]; cf. Fig 3A. The uncontrolled model of the section **Uncontrolled dynamical system** (Fig 1A) is a special case (see below). We interpret the plant to be a rotational joint, so distance is in units of radians; and the control to be a torque, hence in Joules/radian.

The primary rationale for our choice of dynamics and observation model was to show what kinds of computational issues the recurrent, exponential-family harmonium (rEFH) can overcome—issues which it must overcome if it is to be a good model for the way cortex learns to solves the problem. In particular, it might appear that the rEFH can learn relationships only between its current inputs and the previous ones, since its recurrent inputs are from the previous time step only (see Fig 9A). Therefore, we let the inputs report position only, but make the (hidden) dynamics second-order: velocity, as well as position, depends on previous position and velocity. If the rEFH can learn to associate only current and previous inputs, it can learn only first-order dynamics from these data. Furthermore, to clearly distinguish models that have learned second-order dynamics from those that have learned only a first-order approximation, we let the true dynamics be a (damped) oscillator (first-order systems cannot oscillate). Although the demonstration is in terms of positions and velocities, the point is more general: if the rEFH can learn second-order dynamics from position reports, it can learn higher temporal dynamics from lower-order data more generally.

The controlled, single-joint limb obeys:

$$p\left(\theta_{t+1}|\theta_{t},u_{t}^{}\right)=N\left(A\theta_{t}+bu_{t}^{}+\mu_{\theta },\Sigma_{\theta }\right),$$ (1)

where the vector random variable **Θ**_*t*_ consists of angle and angular velocity. The control signal (torque) has itself first-order dynamics:

$$p\left(u_{t+1}^{}|u_{t}^{}\right)=N\left(\alpha u_{t}^{}+\mu_{u},\sigma_{u}^{2}\right),$$ (2)

making the combined system third-order. The initial state and control are also normally distributed:

$$p\left(\theta_{0},u_{0}^{}\right)=N\left(\nu_{0},{ϒ}_{0}\right).$$ (3)

The current (time *t*) joint position and control are noisily encoded in the spike counts of populations of neurons, whose Gaussian-shaped tuning curves (*f*_*i*_) smoothly tile their respective spaces, proprioceptive (angle) and control (torque). Spike counts are drawn from (conditionally) independent Poisson distributions:

$$p\left(r_{t}^{\theta }|\theta_{t},{g_{}^{}}_{t}^{\theta }\right)=\prod_{i}\text{Pois}\left[r_{i,t}^{\theta }|{g_{}^{}}_{t}^{\theta }f_{i}\left(C\theta_{t}\right)\right],\;p\left(r_{t}^{u}|u_{t}^{},{g_{}^{}}_{t}^{u}\right)=\prod_{i}\text{Pois}\left[r_{i,t}^{u}|{g_{}^{}}_{t}^{u}\;f_{i}\left(hu_{t}^{}\right)\right],$$ (4)

with *C* = [1 0] and *h* = 1. Here the *g*_*t*_ are “gains,” scaling factors for the mean spike count [2, 4]. Because the signal-to-noise ratio increases with mean for Poisson random variables, these gains essentially scale (linearly) the reliability of each population. Therefore, in order to model instant-to-instant changes in sensory reliability, the gains of each population were chosen independently and uniformly:

$$p\left({g_{}^{}}_{t}^{\theta }\right)=U(6.4,9.6),\;p\left({g_{}^{}}_{t}^{u}\right)=U(6.4,9.6).$$ (5)

Since the discrete time interval for a single draw from Eq. 4 is 0.05 s (see below), these gains correspond to *maximal* firing rates between 130 and 192 spikes/second, reasonable rates for neurons in cortex. The joint distribution of the states, controls, their observations, and the gains is the product of Eqs 1–5, multiplied across all time.

In accordance with the broad tuning of higher sensory areas, the “standard deviation,” *σ*_tc_, of the tuning curves,

$$f_{i}\left(x\right)=\text{exp}\{-\frac{{\left(x-\xi_{i}\right)}^{2}}{2\sigma_{\text{tc}}^{2}}\},$$

was chosen so that the full-width at half maximum is one-sixth of the space of feasible joint angles/torques, for all preferred stimuli *ξ*_*i*_. However, joints and torques can in fact leave these “feasible spaces”: Although the system was designed to be stable (eigenvalues of the state-transition matrix are within the unit circle), trajectories are nevertheless unbounded, since the input noise is unbounded (normally distributed). We chose not to impose hard joint and torque limits, because this would make the dynamics nonlinear, vitiating the optimality calculations. Instead, stimuli beyond the feasible space simply “wrap” onto the opposite side of encoding space; that is, each population tiles its corresponding stimulus *modulo* the length of its feasible space.

But for the dynamical systems on which model performance was tested, parameters were chosen to make wrapping unlikely (but cf. the “no-spring” model described below). In particular, we used the discrete-time approximation to a damped harmonic oscillator, i.e., $m\ddot{\theta }+c\dot{\theta }+k\theta =u$:

$$A=[\begin{array}{cc} 1 & \Delta \\ -\frac{k}{m}\Delta & 1-\frac{c}{m}\Delta \end{array}],\;b=[\begin{array}{c} 0\\ \frac{\Delta }{m} \end{array}],$$

with moment of inertia *m* = 5 J⋅ s^2^/rad^2^, viscous damping *c* = 0.25 J⋅ s/rad^2^, ideal-spring stiffness *k* = 3 J/rad^2^, and sampling interval Δ = 0.05 s. This makes the system stable and underdamped (oscillatory). The control decay, *α*, in Eq 2 was set to 0.9994, making the dynamics close to a random walk, but mildly decaying towards zero.

These parameters and the noise variances were chosen so that the system could not be well approximated by a lower-order one—i.e., so that the uncontrolled and controlled systems were “truly” second- and third-order (respectively). This was accomplished by ensuring that the Hankel singular values [14] for the system, with output matrix *C* = [1 0] and input matrix set by the noise variances, were within one order of magnitude of each other; that is, ensuring that the transfer function from noise to joint angle had roughly equal power in all modes. For the uncontrolled system, this was achieved with Σ_*θ*_ = diag([5e-7, 5e-5]); for the controlled system, Σ_*θ*_ = diag([5e-5, 1e-6]) and $\sigma_{u}^{2}=7.5\text{E}-4$. While this last choice of noise is large enough to ensure that the control’s contribution to the dynamics is significant, it is also small enough to keep wrapping rare. This facilitates the comparison between the benchmark models (see below), which are acquired from non-wrapped trajectories, and the rEFH, which learns from sensory inputs with periodic tuning curves. That is, for fast enough trajectories on a circle, the dynamics would no longer be locally linear, and the learning and filtering tasks no longer comparable.

The only other difference between the uncontrolled and controlled dynamical systems was that the former had, of course, no control signal (or simply **b** = **0**) and no control observations (efference copy). For all models, the bias terms were set to zero: ***μ***_*θ*_ = **0** and *μ*_*u*_ = 0.

The initial positions for all trajectories were drawn from a uniform distribution across joint space (shoulder *θ* ∈ [−*π*/3, *π*/3] radians; Fig 1C), up to a margin of 0.05 radians from the joint limits (to discourage state transitions out of the feasible space); for EM learning (see below), this was treated as an infinite-covariance Gaussian centered in the middle of joint space. The initial velocity and initial control were normally distributed very tightly about zero, with a standard deviation of $\sqrt{5}\text{E}-5$ (rad/s and J/rad, resp.). Hence ***ν***_0_ = **0**, **Υ**_0_ = diag([∞, 5e-10, 5e-10]). The range (modulus) of feasible controls is *u* ∈ [−1.25, 1.25] J/rad.

For the receptive-field (RF) analyses, we used a third dynamical system. In the harmonic oscillator, whether driven or undriven, the non-zero stiffness (*k* above) couples velocity to position, making high speeds and far-from-zero positions unlikely to co-occur. This makes the RF analysis unreliable in the “corners” of position-velocity space, and the overall velocity-encoding harder to interpret. For the analyses presented in Figs 5, 7 and 8, therefore, we trained a (third) rEFH on a simplified version (“no-spring”) of the uncontrolled dynamics, setting the spring constant to zero (eliminating oscillations). To encourage full exploration of the space, the variance of the state-transition noise was also increased by a factor of 50. The more and less autocorrelated variants of Fig 5D were created by simply scaling up or down the damping coefficient: from left to right, *c* = 0.25/4, 0.25/2, 0.25, 0.25 * 2, 0.25 * 4. For completeness, we nevertheless include, in the Supplement, the harder-to-interpret RF analyses for the rEFH trained on the (undriven) harmonic oscillator (S3 Text).

### The recurrent, exponential-family harmonium (rEFH)

The network is very similar to that in [4], but we repeat the description here briefly. The harmonium is a generalization of the restricted Boltzmann machine (RBM) beyond Bernoulli units to other random variables in the exponential family [3]. That is, it is a two-layer network with full interlayer connections and no intralayer connections, which can be thought of as a Markov random field (undirected graphical model) or as a neural network. In our implementation (see Figs 1B and 3B), hidden units (turquoise, **Z**_*t*_) and recurrent units (dark turqoise, **Z**_*t*−1_) are binary (spike/no spike), and the “proprioceptive” (orange, $R_{t}^{\theta }$) and “efference-copy” (purple, $R_{t}^{u}$) populations are non-negative integers (spike counts). For all networks, the number of recurrent units is the same as the number of downstream or “hidden” units, because recurrent units at time *t* carry the activities of the hidden units at time *t* − 1—making the harmonium recurrent through time (rEFH). We chose *N*_hid_ = *N*_recurrent_ = 240 for the network trained on the uncontrolled system, and *N*_hid_ = *N*_recurrent_ = 180 for the controlled system. We used fifteen proprioceptive units (*N*_prop_) and, for the network trained on the controlled system, fifteen efference-copy units (*N*_efcp_), so the total number of “observed” (or “input”) variables was 255 = *N*_recurrent_ + *N*_prop_ for the uncontrolled model and 210 = *N*_recurrent_ + *N*_prop_ + *N*_efcp_ for the controlled model.

During training and testing, the layers of the rEFH reciprocally drive each other, yielding samples from the following distributions:

$${Z_{}^{}}_{t}∼q\left({z_{}^{}}_{t}|{z_{}^{}}_{t-1},r_{t}^{\theta },r_{t}^{u}\right)=\prod_{i}^{N_{\text{hid}}}\text{Bern}[{\left\{{z_{}^{}}_{t}\right\}}_{i}|\sigma ({\left\{W_{\text{fb}}{z_{}^{}}_{t-1}+W_{\text{prop}}r_{t}^{\theta }+W_{\text{ctrl}}r_{t}^{u}+b_{\text{hid}}\right\}}_{i})]$$ (6a)

$${Z_{}^{}}_{t-1}∼q\left({z_{}^{}}_{t-1}|{z_{}^{}}_{t}\right)=\prod_{i}^{N_{\text{hid}}}\text{Bern}[{\left\{{z_{}^{}}_{t-1}\right\}}_{i}|\sigma ({\left\{W_{\text{fb}}^{\text{T}}{z_{}^{}}_{t}+b_{\text{fb}}\right\}}_{i})]$$ (6b)

$$R_{t}^{\theta }∼q\left(r_{t}^{\theta }|{z_{}^{}}_{t}\right)=\prod_{i}^{N_{\text{prop}}}\text{Pois}[{\left\{r_{t}^{\theta }\right\}}_{i}|\text{exp}({\left\{W_{\text{prop}}^{\text{T}}{z_{}^{}}_{t}+b_{\text{prop}}\right\}}_{i})]$$ (6c)

$$R_{t}^{u}∼q\left(r_{t}^{u}|{z_{}^{}}_{t}\right)=\prod_{i}^{N_{\text{efcp}}}\text{Pois}[{\left\{r_{t}^{u}\right\}}_{i}|\text{exp}({\left\{W_{\text{efcp}}^{\text{T}}{z_{}^{}}_{t}+b_{\text{efcp}}\right\}}_{i})],$$ (6d)

which corresponds to Gibbs sampling from the joint distribution represented by the harmonium, $q(z_{t},z_{t-1},r_{t}^{\theta },r_{t}^{u};W,b)$. The letter *q* is used for the probability density function assigned by the rEFH to distinguish it from the true distribution over the observed variables, $p(r_{t}^{\theta },r_{t}^{u})$. Here the notation {**x**}_*i*_ means the *i* ^th^ element of the vector **x**; the matrices *W* and vectors **b** are the synaptic connection strengths (“weights”) and biases, respectively; and the neural nonlinearities, the logistic (*σ*(*x*) = 1/(1 + *e* ^−*x*^)) and exponential funtions, were chosen to produce means for each distribution that are in the appropriate interval ([0, 1] and ${\mathbb{R}}_{+}$, resp.). The entire procedure is depicted graphically in Fig 9A.

#### Training

Although the ultimate goal of training is to make the network able to solve the filtering problem, this is achieved indirectly by making the harmonium a good model for the data on which it was trained. That is, the harmonium should assign probability (*q*) to the observed data (**Y**) that matches the probability with which they actually appear (*p*); in short, the goal is to achieve:

$$q\left(y_{}^{};W,b\right)=p\left(y_{}^{}\right),$$ (7)

equality between the “model distribution” and “data distribution,” by adjustment of the weights and biases of the network. In our case, **Y** = [**Y**_0_, ⋯, **Y**_*T*_], a collection of observations across time, where intuitively the observations at time *t* are the responses of the proprioceptive and efference-copy populations, $Y_{t}=\left[R_{t}^{\theta },\;R_{t}^{u}\right]$. However, these random variables are not independent across time; that is, $p(r_{0}^{\theta },r_{0}^{u},\ldots ,r_{T}^{\theta },r_{T}^{u})\neq \prod_{t}p(r_{t}^{\theta },r_{t}^{u})$. In order, then, to make possible incremental training—weight changes without first collecting population responses for all time, [0, …, *T*]—we train on the augmented observation vector:

$$Y_{t}=[Z_{t-1},R_{t}^{\theta },R_{t}^{u}],$$ (8)

where **Z**_*t*−1_ are the hidden-unit activities at the previous time step. Intuitively, the addition of these recurrent activities renders the data independent because they recursively accumulate all the information contained in their inputs [9].

Weight changes are made proportional to the approximate gradient of a function (“one-step contrastive divergence,” CD_1_) that has Eq 7 at its minimum [12, 13]. In exponential family harmoniums, following this gradient is particularly simple: Stimuli in the world drive the input populations (**y**, Eq 4), which drive the hidden units (**z**, Eq 6a), which reciprocally drive the input populations ($\hat{y}$, Eqs 6b, 6c and 6d), which drive the hidden units once more ($\hat{z}$, Eq 6a); after which parameters are changed according to:

$$\Delta W\propto yz^{\text{T}}-\hat{y}{\hat{z}}^{\text{T}},\;\Delta b_{y}\propto y-\hat{y},\;\Delta b_{z}\propto z-\hat{z}.$$ (9)

Note that the learning rule is local and Hebbian (correlational). The entire procedure amounts to taking a full step of Gibbs sampling in a Markov chain that has been initialized at a vector sampled from the “data distribution” *p*, and then changing weights so as to penalize the network for drifting away from the data distribution. In practice, we depart from Eq 9 by using “momentum” and “weight decay” [15], as is standard in neural-network training. Our choice of momentum and decay make this equivalent to low-pass filtering the learning signal (the right-hand sides of the equations) with an overdamped second-order system before making weight changes. Biologically, it corresponds to changes in synaptic strength having their own intrinsic dynamics.

Training took place in “epochs.” Data in each epoch consisted of 40,000 vectors: 40 trajectories of 1000 time steps apiece, each vector consisting of the current sensory response (proprioceptive and efference-copy) and the previous hidden-unit activities (“recurrent”; see Fig 3B). On the initial time step, the recurrent units were set to all zeros and drove no weight changes. In order to accelerate convergence, and although biological implausible, weight changes were made on “minibatches” of 40 input vectors, each of which corresponded to the same time point, but from the 40 different trajectories. Fresh data (40 new trajectories) were generated every five epochs. Learning rates (*ϵ*) also decayed across epochs. For the rEFH trained on an uncontrolled dynamical system, the total number of epochs was 120, and the decay was exponential: for the *k* ^th^ epoch, $\epsilon_{k}^{-1}=1.1^{k}\epsilon_{0}^{-1}$. For the case of controlled dynamics, the network was trained for 1200 epochs, with the reciprocal learning rate growing according to a sigmoidal function: $\epsilon_{k}^{-1}=\;\left(\frac{1000}{1+\text{exp}\{-k/8+7.5\}}\;+\;\frac{1}{2}\right)\epsilon_{0}^{-1}$ . (The numbers were chosen so that the sigmoid approximately matches the exponential growth for the first 120 epochs, although their exact values are not critical.)

#### Testing

Filtering was tested on a new set of 40 trajectories (40,000 vectors). At each time step, the current “sensory” (proprioception and efference copy) and recurrent responses were fed forward to the hidden layer of the network, as in training. Unlike training, however, no samples were taken from this vector of means; instead, the real-valued vector was itself returned as the recurrent response. This is equivalent to taking several (∼ 15) samples and averaging [4]; the means themselves were used to simplify presentation of the results, since they correspond to the maximum achievable performance of the network.

Formally, the solution to the filtering problem is the optimal posterior distribution over the current stimulus location, given all the observations up to this point in time: $p(\theta_{t}|r_{0}^{\theta },\ldots ,r_{t}^{\theta },r_{0}^{u}{,}_{\ldots }^{,}r_{t}^{u})$. For the controlled dynamical system, we also ask about the posterior distribution over the controls, $p(u_{t}|r_{0}^{\theta },\ldots ,r_{t}^{\theta },r_{0}^{u},\ldots ,r_{t}^{u})$, since they are observed only noisily at each time step. We discuss optimality below, but note here that in our case these distributions are Gaussian, so their only non-zero cumulants are mean and covariance. Generically, proving optimality of the harmonium would require showing that both these cumulants can be recovered from its hidden units at every point in time; but in the present case it is only necessary to decode the posterior mean, since it is impossible for the network to keep track of the mean without also keeping track of the covariance: incorrect estimates of the latter would result in mis-weighting of the relative reliability of current sensory information and current filter estimate, resulting in suboptimal inference of the mean at the next time step.

Decoding the rEFH’s hidden units exploits a trick [4]. The representational space of the hidden units is obscure; therefore, the hidden unit activities (a real-valued vector) are passed back down through the network, i.e. into the space of the inputs. Here, the optimal decoding scheme is known: it is the center of mass of each noisy hill of activity [4]. This decoder was applied to hidden units at each time step, for each of the 40 testing trajectories, from which errors from the actual joint angle and control input were computed.

### The optimal filtering distribution

For the graphical models in Figs 1A and 3A, the solution to the filtering problem can be assimilated to a variant on the Kalman filter, and therefore computed in closed form. This is because, although the emission $p(r_{t}^{\theta }|\theta_{t})$ is not a Gaussian distribution over $r_{t}^{\theta }$, it is a Gaussian function of ***θ***_*t*_ [4, 7] (i.e., the likelihood is an unnormalized Gaussian over ***θ***_*t*_)—or more precisely, of *C* ***θ***_*t*_, with *C* the observation matrix (see Eq 4)—and this is the critical requirement for the derivation of Kalman’s recursive solution. The resulting modification is small: Where the emission variance and the (Gaussian-distributed) emission appear in the standard KF equations, we substitute, respectively, the scaled tuning-curve width, $\sigma_{\text{tc}}^{2}/\sum_{i}\;r_{i}^{\theta }$, and the center of mass of the population response, $\sum_{i}\xi_{i}r_{i}^{\theta }/\sum_{i}r_{i}^{\theta }$ [16].

The same applies, *mutatis mutandis*, to the controls. In fact, the “controlled” case provides no additional complexity, since it corresponds to an uncontrolled third-order system (since the control has its own dynamics) whose state **X**_*t*_ is the concatenation of **ϴ**_*t*_ and *U*_t_:

$$p\left(x_{t+1}^{}|x_{t}^{}\right)=N\left(\Gamma x_{t}^{}+\mu_{x},\Sigma_{x}\right),$$ (10)

with

$$\Gamma :=[\begin{array}{ccc} 1 & \Delta & 0\\ -\frac{k}{m}\Delta & 1-\frac{c}{m}\Delta & \frac{\Delta }{m}\\ 0 & 0 & \alpha \end{array}],\;\mu_{x}:=[\begin{array}{c} \mu_{\theta }\\ \mu_{u} \end{array}],\;\Sigma_{x}:=[\begin{array}{cc} \Sigma_{\theta } & 0\\ 0 & \sigma_{u}^{2} \end{array}].$$

In both cases, then, the posterior (filtering) distribution over the state is always Gaussian, so at every time step, one computes the posterior mean and covariance, which can be expressed in terms of the filtering distribution at the previous time step, and of the current sensory information. A full derivation appears in S1 Text.

Eq 10 ignores some independence statements asserted by the graph of Fig 3A. In fact, an EM algorithm that accounts for them can be derived; but in our experiments, this algorithm does not achieve superior results to the “agnostic” version that tries to learn unconstrained versions of Γ, ***μ***_*x*_, and Σ_*x*_. Therefore, results for EM^3^ throughout use the unconstrained version of the algorithm.

#### Benchmark models

Error statistics for the rEFH are compared to those from four types of model. It is simplest to think of all four types using the same filtering algorithm—the KF, modified as described to account for the Poisson emissions—but running that filter on different generative models for the observed data ($r_{t}^{\theta },\;r_{t}^{u}$).

- **PROP and EfCp**: In the simplest benchmark model, joint-angle (PROP) and control (EfCp) estimates are made simply via the center of mass on the current “sensory” population. This is the optimal decoder for populations of smoothly tiled, Gaussian-tuned, Poisson neurons [17], under the assumption of independence through time (no dynamics). It can also be thought of as a Kalman filter applied to a generative model with infinite state-transition covariance, Σ_*x*_. (This severs the horizontal connections in Figs 1A and 3A.)
- **OPT**: The “optimal” model runs the KF on the true generative model for the data, i.e., using the true parameters, $\left\{A,b,\;C,\;\alpha ,\;h,\sum_{\theta },\sigma_{u}^{2},\nu_{0},{ϒ}_{0}\right\}$.
- **EM^*n*^**: The rEFH was not, of course, given its parameters, but had to learn them, and only from the noisy population responses, $R_{0}^{\theta },\ldots ,R_{T}^{\theta }$, $R_{0}^{u},\ldots ,R_{T}^{u}$. A useful point of comparison, then, is the performance of a (Kalman) filter that has learned those parameters from the same noisy data, but following a learning procedure that is known to be optimal. For our linear, time-invariant systems, such learning rules can be derived: they are an implementation of expectation-maximization (EM), an algorithm that guarantees convergence to at least local optima. Applying EM to linear dynamical systems requires both a forward pass through the data, filtering, *and* a backward pass for smoothing, i.e., computing the probability of the hidden state given the observations *for all time*. These equations are derived in S1 Text. Likewise, the algorithm must be told the order (number of states) of the latent dynamical system. Which order it was told is indicated by a superscript (e.g., EM^2^). We emphasize that access to the backward pass of observations, and knowledge of the order of the latent dynamics, are advantages the EM-trained models (EM^*n*^) enjoyed over the harmonium.
- **OBS**: For the controlled system (Controlled dynamical system), one would also like to know how useful knowledge of the controls is to inference of the joint angle. The optimal model that ignores controls, again under the assumption of linear dynamics, can be constructed by training on fully observed data, where the model parameters $\left\{A,\;C,\sum_{\theta },\nu_{0},{ϒ}_{0}\right\}$ are learned from **ϴ**_0_, …, **ϴ**_*T*_, $R_{0}^{\theta },\ldots ,R_{T}^{\theta }$, essentially via linear regression (see S1 Text). This corresponds to using the generative model of Fig 1A, even though the true data were generated by the model of Fig 3A. OBS is therefore suboptimal, but tells us how much suboptimality accrues by ignoring the controls. (We fit the parameters of OBS, rather than providing them, because the fit model can actually outperform one based on the true dynamical-system parameters. This is because OBS can compensate to some extent for the missing controls by overestimating the state-transition noise. If the model were forced to use the true state-transition noise, but still assume zero control input, it would be worse at explaining state transitions.)

The benchmark models also enjoyed another advantage over the rEFH. The sufficient statistics of the emission for the state are the population center of mass and the scaled tuning-curve width (or simply the scaling factor), as alluded to above. These were given to the benchmark models, rather than learned. (In the standard model, linear-Gaussian emissions with fixed variance, learning that variance with EM is straightforward [18]. For our more complicated emission model, it is not, which is why we decided simply to provide it to the benchmark models.) The harmonium, on the other hand, had to learn what to do with the vectors of raw spike counts, **r** ^*θ*^, **r** ^*u*^.

Different training runs, like different testing sets, will yield slightly different models. Thus, for each type of model to be trained, including the rEFH, we selected the best of 20 different networks, each trained from scratch with random initialization. Then we repeated this procedure itself twelve times, and from these twelve tokens of each model type, generated the error bars for the MSEs in Figs 1E, 3D and 3F. Each of the twelve tokens of a particular model type was tested on a different testing set, but the *same* testing set was used for matching tokens of different types (so, e.g., the fourth rEFH token was tested on the same data as the fourth EM^2^ token).

### Tuning analysis

In the section **Learned receptive fields and connectivity**, in order to determine how the network has learned to solve the filtering problem, we sort hidden units by their “preferred” lags and “preferred” angles. These were computed as follows. First, we generated a new set of 40 trajectories of 1000 time steps apiece. Then we computed hidden-unit mean activities, i.e., their probability of firing (these are the same quantity because the hidden units are conditionally Bernoulli random variables). Angular positions for all 40000 time points were then discretized into 30 bins of uniform width spanning the feasible joint space.

For each hidden unit, the following calculation was then performed. First, the empirical mutual information (MI) was computed, according to the standard formula [19], between the two discrete random variables: the discretized position (30 bins) and the binary (spike/no spike) hidden-unit response. Next, to reject spurious MI (which will anyway be rare, given the number of data), for each of 20 reshuffles, the unit’s response was shuffled in time and the MIs recalculated. If the unit’s unshuffled MI fell below the 95th percentile of its shuffled MIs, the unshuffled MI was set to zero. The entire procedure was then repeated with the response time-shifted forward by one step, for each of 40 steps. Finally, the “preferred” lag was selected to be the time shift for which MI was maximized. These were used to sort the receptive fields in Figs 5A and 5B, 7 and 8B.

For each unit, a “lagged” tuning curve can be constructed by considering its mean responses to past (discretized) stimuli; in particular, to stimuli at that unit’s *preferred* lag. These are the curves plotted as a heat map in Fig 5C, where they have been sorted by the locations of the tuning curves’ peaks. The same locations were used to sort the weight matrix in Fig 8A. Inverting the process, one can ask how well these tuning curves explain the receptive fields in the space of non-delayed position and velocity (Fig 5A): apply each tuning curve to each of the 40000 stimuli, delay the responses by the units’ preferred lags, and then compute receptive fields with these responses. This is how Fig 5B was constructed.

Finally, comparing the distribution of preferred lags (Fig 5D) to the autocorrelation of the stimulus required computing the autocorrelation of a circular variable (angle). We used the angular-angular correlation measure given by Zar [20].

## Supporting Information

S1 Text (PDF)

S2 Text (PDF)

S3 Text (PDF)

S1 Fig (PDF)

S2 Fig (PDF)

S4 Text (PDF)

S3 Fig (PDF)

S1 Bibliography (PDF)
