# Supplementary material for: Learning to Estimate Dynamical State with Probabilistic Population Codes
Source: PLoS Comput Biol. 2015 Nov 5;11(11):e1004554. doi: 10.1371/journal.pcbi.1004554 (PMC4634970; doi:10.1371/journal.pcbi.1004554)
Supplement: S1 Text — (PDF) [file pcbi.1004554.s001.pdf]

S1 Text: LTI control systems with probabilistic-population-code emissions

We show that state estimation in the graphical models of Fig. 1A and Fig. 3A can be assimilated to the standard linear dynamical system with linear-Gaussian emissions. This implies that the filtering (and smoothing) can be performed with Kalman's equations, and that the model parameters can be learned with an expectation-maximization (EM) algorithm. A version of this derivation appears in the supporting material in reference [7] but we include ours here for completeness. We emphasize that the point of this assimilation is that it allows us to compute what "optimal" inference and learning are for the generative models of Fig. 1A and Fig. 3A, providing a useful point of comparison for the performance of our neural-network model (rEFH). But the rEFH is not in principle limited to learning generative models that can be assimilated to the standard case.

Inference. The critical fact for the assimilation is that the derivation of the Kalman filter does not require the emission ($\mathbf{Y}_t$) probabilities to be normal distributions over linear functions of the state ($\mathbf{X}_t$); i.e., $\Pr(\mathbf{y}_t|\mathbf{x}_t) = \mathcal{N}(M\mathbf{x}_t, \Sigma)$, for some matrix M—although this is sufficient. Instead, it requires that the *likelihood* be expressible as a *Gaussian function* of the state; i.e., $\Pr(\mathbf{y}_t|\mathbf{x}_t) \propto \exp\{-\mathbf{x}_t{}^{\mathrm{T}} M \mathbf{x}_t + \mathbf{v}^{\mathrm{T}}\mathbf{x}_t\}$, where the (omitted) proportionality constant is independent of $\mathbf{x}_t$ and M is symmetric positive definite. It has been shown elsewhere (e.g., [2] or [16]) that, for Gaussian-tuned, Poisson neurons that smoothly tile the space of stimuli—or, more precisely, $C\boldsymbol{\theta}_t$—the likelihood of those stimuli $\boldsymbol{\theta}_t$ (cf. Eq. 4) is approximately:

$$\begin{aligned}\Pr(\mathbf{r}_t^\theta|\boldsymbol{\theta}_t, g^\theta) &= \prod_i \mathrm{Pois}[r_{i,t}^\theta | g_t^\theta f_i(C\boldsymbol{\theta}_t)] \\ &\approx \alpha(\mathbf{r}_t^\theta, g^\theta) \exp\left\{ -\frac{1}{2}(C\boldsymbol{\theta}_t - \boldsymbol{\psi}(\mathbf{r}_t^\theta))^{\mathrm{T}} \bar{\Sigma}(\mathbf{r}_t^\theta)^{-1} (C\boldsymbol{\theta}_t - \boldsymbol{\psi}(\mathbf{r}_t^\theta)) \right\},\end{aligned} \tag{S1}$$

for some function α independent of $\boldsymbol{\theta}$, as well as:

$$\bar{\Sigma}(\mathbf{r}_t^\theta) = \frac{\Sigma_{\mathrm{tc}}}{\sum_i r_{i,t}^\theta}, \tag{S2}$$

the scaled tuning-curve covariance; and

$$\boldsymbol{\psi}(\mathbf{r}_t^\theta) = \frac{\sum_i \boldsymbol{\xi}_i r_{i,t}^\theta}{\sum_i r_{i,t}^\theta}, \tag{S3}$$

the center of mass of the population response, where $\boldsymbol{\xi}_i$ is the preferred stimulus of neuron i. Here, g, the "gain," is the instantaneous reliability of the population, which simply scales the heights of all the tuning curves, and the f_i are the Gaussian tuning curves. For generality, we allow multi-dimensional tuning curves, which is why tuning-curve "width" (in units of variance) is specified with a matrix, Σ_{tc}. The approximation becomes more accurate as the number of neurons increases.

Now consider *two* such populations, with responses $\mathbf{R}_t^\theta$ and $\mathbf{R}_t^u$. If they are independent conditioned on the stimuli they encode, $\boldsymbol{\theta}_t$ and $\mathbf{u}_t$, then:

$$\begin{aligned}\Pr(\mathbf{r}_t^\theta, \mathbf{r}_t^u|\boldsymbol{\theta}_t, \mathbf{u}_t, g^\theta, g^u) &= \prod_i \mathrm{Pois}[r_{i,t}^\theta | g_t^\theta f_i(C\boldsymbol{\theta}_t)] \prod_j \mathrm{Pois}[r_{j,t}^u | g_t^u f_j(H\mathbf{u}_t)], \\ &\approx \alpha(\mathbf{r}_t^\theta, \mathbf{r}_t^u, g_t^\theta, g_t^u) \exp\left\{ -\frac{1}{2}(\Lambda\mathbf{x}_t - \boldsymbol{\psi}_t)^{\mathrm{T}} \bar{\Sigma}_t^{-1} (\Lambda\mathbf{x}_t - \boldsymbol{\psi}_t) \right\},\end{aligned} \tag{S4}$$

where:

$$\mathbf{x}_t := \begin{bmatrix}\boldsymbol{\theta}_t \\ \mathbf{u}_t\end{bmatrix}, \qquad \Lambda := \begin{bmatrix} C & 0 \\ 0 & H\end{bmatrix}, \qquad \boldsymbol{\psi}_t := \begin{bmatrix}\boldsymbol{\psi}(\mathbf{r}_t^\theta) \\ \boldsymbol{\psi}(\mathbf{r}_t^u)\end{bmatrix}, \qquad \bar{\Sigma}_t := \begin{bmatrix}\bar{\Sigma}(\mathbf{r}_t^\theta) & 0 \\ 0 & \bar{\Sigma}(\mathbf{r}_t^u)\end{bmatrix}.$$

Eq. S4 has the same form as Eq. S1, so we derive the result for both at once in terms of $\mathbf{x}_t$, Λ, ψ_t, and $\bar{\Sigma}_t$, and a generic data vector $\mathbf{y}_t := \begin{bmatrix}\mathbf{r}_t^\theta \\ \mathbf{r}_t^u\end{bmatrix}$. To apply the result to the one-population (no efference copy) case, one simply lets:

$$\mathbf{x}_t := \boldsymbol{\theta}_t, \qquad \Lambda := C, \qquad \boldsymbol{\psi}_t := \boldsymbol{\psi}(\mathbf{r}_t^\theta), \qquad \bar{\Sigma}_t := \bar{\Sigma}(\mathbf{r}_t^\theta), \qquad \mathbf{y}_t := \mathbf{r}_t^\theta.$$

The likelihood of Eq. S4 enters into the derivation of the Kalman filter via the "measurement update." Given the cumulants $(\hat{\mathbf{x}}_{t+1|t}, \Upsilon_{t+1|t})$ of the "time update," where $\Pr(\mathbf{x}_{t+1}|\mathbf{y}_{0:t}) = \mathcal{N}(\hat{\mathbf{x}}_{t+1|t}, \Upsilon_{t+1|t})$, one updates the posterior expectation to condition on the next (i.e., $(t+1)^{\text{th}}$) observation:

$$
\begin{aligned}
\Pr(\mathbf{x}_{t+1}|\mathbf{y}_{0:t+1}) &\propto \Pr(\mathbf{y}_{t+1}|\mathbf{x}_{t+1}) \Pr(\mathbf{x}_{t+1}|\mathbf{y}_{0:t}) \\
&\propto \exp\left\{ -\frac{1}{2}(\Lambda\mathbf{x}_{t+1} - \psi_{t+1})^{\mathrm{T}}\bar{\Sigma}_{t+1}^{-1}(\Lambda\mathbf{x}_{t+1} - \psi_{t+1}) \right\} \mathcal{N}(\hat{\mathbf{x}}_{t+1|t}, \Upsilon_{t+1|t}) \\
&\propto \exp\left\{ -\frac{1}{2}(\Lambda\mathbf{x}_{t+1} - \psi_{t+1})^{\mathrm{T}}\bar{\Sigma}_{t+1}^{-1}(\Lambda\mathbf{x}_{t+1} - \psi_{t+1}) \right. \\
&\qquad \left. -\frac{1}{2}(\mathbf{x}_{t+1} - \hat{\mathbf{x}}_{t+1|t})^{\mathrm{T}}\Upsilon_{t+1|t}(\mathbf{x}_{t+1} - \hat{\mathbf{x}}_{t+1|t}) \right\} \\
&\propto \exp\left\{ -\frac{1}{2}(\mathbf{x}_{t+1} - \hat{\mathbf{x}}_{t+1|t+1})^{\mathrm{T}}\Upsilon_{t+1|t+1}^{-1}(\mathbf{x}_{t+1} - \hat{\mathbf{x}}_{t+1|t+1}) \right\} \\
\implies \Pr(\mathbf{x}_{t+1}|\mathbf{y}_{0:t+1}) &\approx \mathcal{N}(\hat{\mathbf{x}}_{t+1|t+1}, \Upsilon_{t+1|t+1});
\end{aligned}
\tag{S5}
$$

where:

$$
\begin{aligned}
\Upsilon_{t+1|t+1} &:= \left(\Lambda^{\mathrm{T}}\bar{\Sigma}_{t+1}^{-1}\Lambda + \Upsilon_{t+1|t}\right)^{-1}, \\
\hat{\mathbf{x}}_{t+1|t+1} &:= \Upsilon_{t+1|t+1}(\Lambda^{\mathrm{T}}\bar{\Sigma}_{t+1}^{-1}\psi_{t+1} + \Upsilon_{t+1|t}^{-1}\hat{\mathbf{x}}_{t+1|t}).
\end{aligned}
$$

Line one follows from the independence statements of the graph (Fig. 1A or Fig. 3A). The fourth line results from completing the square in the exponent. The final line follows because the (omitted) proportionality constants are all independent of $\mathbf{x}_{t+1}$.

Since the time updates:

$$
\Pr(\mathbf{x}_{t+1}|\mathbf{y}_{0:t}) = \int_{\mathbf{x}_t} \Pr(\mathbf{x}_{t+1}|\mathbf{x}_t) \Pr(\mathbf{x}_t|\mathbf{y}_{0:t}) \,\mathrm{d}\mathbf{x}_t \tag{S6}
$$

are just like the standard Kalman-filter updates, they retain the Gaussian posterior distribution, and thus the posterior is Gaussian for all time (as long as it starts that way, i.e., the initial conditions are normally distributed). Thus inference (estimation) in the models of Fig. 1A or Fig. 3A can be assimilated directly to the Kalman filter on a standard linear, time-invariant system, where the center(s) of mass and scaled tuning covariance(s) play the role of the emission and its covariance, respectively. During training and testing, we supply these directly to the models OPT, OBS, and EM^n; that is, these systems never have to learn the emission covariance, nor the (nonlinear) transformation from firing rates $\mathbf{r}_t^\theta, \mathbf{r}_t^u$ to center of mass.

Learning. Parameter learning is implemented with an EM algorithm [18]. The E-step is a forward filtering pass followed by a (backward) smoothing pass, yielding a posterior distribution over the state for all time, $q(\boldsymbol{\theta}_{0:T}, \mathbf{u}_{0:T}|\mathbf{r}_{0:T}^\theta, \mathbf{r}_{0:T}^u; \phi)$. The M-step consists of solving for the parameters, $\phi = \{\Gamma, \Lambda, \boldsymbol{\mu}_x, \Sigma_x, \boldsymbol{\nu}_0, \Upsilon_0\}$, that maximize $q(\boldsymbol{\theta}_{0:T}, \mathbf{u}_{0:T}|\mathbf{r}_{0:T}^\theta, \mathbf{r}_{0:T}^u; \phi)$ (considered as a likelihood of ϕ), averaged under the observed data. This amounts to a set of linear regressions and the solution of their corresponding normal equations. E and M steps are alternated until the likelihood stops increasing. When the state and control are observed, as in the model OBS, no alternation is required, and one simply solves a single set of normal equations.
