# Supplementary material for: Learning to Estimate Dynamical State with Probabilistic Population Codes
Source: PLoS Comput Biol. 2015 Nov 5;11(11):e1004554. doi: 10.1371/journal.pcbi.1004554 (PMC4634970; doi:10.1371/journal.pcbi.1004554)
Supplement: S2 Text — (PDF) [file pcbi.1004554.s002.pdf]

S2 Text: Filtering vs. multisensory integration

We consider the connection and the differences between filtering (dynamical-state estimation), the computational problem solved this paper, and multisensory integration, which we have investigated in previous work [4] That paper showed that the hidden units of an EFH trained on static data—e.g., a population of proprioceptive units, and a population of visual units, both reporting the location of the hand—will learn to combine optimally the information in those inputs. Put otherwise, the hidden (multisensory) units don't lose information about the underlying stimulus. In state estimation, on the other hand, optimality requires not losing information about the stimulus that was in *all* of the preceding inputs, from the beginning of the trajectory.

The distinction can be made crisply in terms of mutual information. The task in multisensory integration is to ensure that the multisensory activities, $\mathbf{Z}$, have approximately the same mutual information with the underlying stimulus as the two unisensory populations, $\mathbf{R}_1^\theta, \mathbf{R}_2^\theta$, do:

$$\mathcal{I}(\mathbf{Z};\mathbf{\Theta}) \approx \mathcal{I}(\mathbf{R}_1^\theta, \mathbf{R}_2^\theta; \mathbf{\Theta}).$$

The task in state estimation is to ensure that the hidden units at time t have approximately the same mutual information with the stimulus at time t as *all the observations up the present time* do:

$$\mathcal{I}(\mathbf{Z}_t;\mathbf{\Theta}_t) \approx \mathcal{I}(\mathbf{R}_0^\theta, \mathbf{R}_1^\theta, \ldots, \mathbf{R}_t^\theta; \mathbf{\Theta}_t).$$

Had we trained the EFH on all inputs $\mathbf{R}_0^\theta, \mathbf{R}_1^\theta, \ldots, \mathbf{R}_t^\theta$ at once, then the models would be the same (it would be like having t sense modalities). Of course, this would be rather impractical, since it would require augmenting the size of the input vector at every time step! Instead, we showed that it was sufficient to train only on the current data $\mathbf{R}_t^\theta$ and the previous hidden activities, $\mathbf{Z}_{t-1}$.

The fact that this is sufficient can be understood intuitively as follows. Filtering can be thought of as a two step process: first, a "multisensory integration" of the current observation with the filtering distribution at the previous time step—the "measurement update" of Eq. S5; second, the time update, Eq. S6, which essentially adds two random variables. We also showed in our previous work [4] that the EFH can learn to perform such additions, followed by multisensory integrations: the hidden units of the "coordinate-transformation" models of that paper contain the information of random variables computed in this way. This suggest that the hidden units of a model trained on a population representing the filtering distribution and a population encoding sensory information can learn to extract optimally the information in the two: adding random variables and then multisensory-integrating, or equivalently time updating and measurement updating. Then these hidden units can be used as inputs at the next time step.
