# Supplementary material for: Learning to Estimate Dynamical State with Probabilistic Population Codes
Source: PLoS Comput Biol. 2015 Nov 5;11(11):e1004554. doi: 10.1371/journal.pcbi.1004554 (PMC4634970; doi:10.1371/journal.pcbi.1004554)
Supplement: S3 Text — (PDF) [file pcbi.1004554.s003.pdf]

S3 Text: Receptive fields for other models

The receptive fields (RFs) investigated in the main text all came from an rEFH trained on an uncontrolled, second-order, linear dynamical system, which in particular had zero stiffness (and hence did not oscillate). The large majority of RFs in that harmonium took a particular, interesting form: negatively sloped "stripes" in position-velocity space (Fig. 5). These are very well explained by tuning for position alone, but at various time lags (with steeper slopes corresponding to longer delays)—although as a population they do carry velocity information. This led us to predict that in higher cortical areas, especially posterior parietal cortex, such RFs will develop during initial exposure to dynamic stimuli. More generally, we predicted the appearance of RFs that are tuned to past dynamical states of any order (for example, past velocities), as long as the next-order dynamical state (in the example, acceleration) is itself *lawfully* time-varying. In the following subsections we show that such RFs are not peculiar to our choice of dynamical system or observation model, although their prevalence is.

Different dynamics. In Fig. S1, we consider an rEFH trained on the uncontrolled, damped, harmonic oscillator—i.e., one of the networks whose performance was characterized in Fig. 1. The rEFH whose RFs were characterized in Figs. 5 and 8 in the main text had no spring force (and hence no oscillation) and higher noise in state transitions, in order to encourage full exploration of position-velocity space. Unsurprisingly, then, the RFs in Fig. S1A are comparatively sparser, especially in the "corners" of the RFs, since the spring force prevents trajectories from reaching high speeds at distances far from equilibrium. This also results in shallower slopes (cf. Fig. S1A and Fig. 5A) and shorted autocorrelations (cf. Fig. S1D and Fig. 5D). Similarly, since oscillatory trajectories rarely leave the feasible space, the learned RFs in Fig. S1A make fewer cycles.

Nevertheless, the overall picture is qualitatively quite similar to Figs. 5 and 8. Idealized RFs constructed by tuning purely for lagged position (Fig. S1B) still approximate well most of the measured RFs; and this tuning again tiles position space roughly uniformly (Fig. S1C). The distribution of lags follows the autocorrelation of the underlying dynamics (Fig. S1D), as in Fig. 5D, although the units not well characterized by lagged-position tuning (first and last columns of Fig. S1A), being misclassified as maximally or minimally lagged, give rise to spurious peaks at the beginning and end of the histogram. The organized weight matrices likewise look quite similar (cf. Fig. S1E,F and Fig. 8A,B).

Different observation model. In Fig. S2, we examine the RFs of an rEFH trained on (noisy) reports of *velocity* as well as position. Thus, the "sensory population" consists of two populations of 15 neurons apiece, one reporting joint angle and the other reporting angular velocity. The dynamics are again those of Fig. 5 (no stiffness). We again consider the RFs in position-velocity (y-x) space. In contrast to the RFs investigated above (Figs. 5 and S1), some units here are clearly tuned to pure velocity (vertical stripes in Fig. S2A). Nevertheless, a significant fraction of the RFs appear again to be position-lagged units, as in the previous figures. Some of these are shown enlarged in Fig. S2B. Performance of this model is also close to optimal, as usual (Fig. S2C).

Since the dynamics are (only) second-order, we do not expect to find units tuned to past *velocities*, although for more general dynamics we do indeed expect this to be the case. Yet, we emphasize, even for stimuli with second-order dynamics and direct reports of velocity, lagged tuning appears. This suggests something of the generality of this encoding scheme.
