# Supplementary material for: Learning to Estimate Dynamical State with Probabilistic Population Codes
Source: PLoS Comput Biol. 2015 Nov 5;11(11):e1004554. doi: 10.1371/journal.pcbi.1004554 (PMC4634970; doi:10.1371/journal.pcbi.1004554)
Supplement: S1 Fig — (PDF) [file pcbi.1004554.s004.pdf]

S1 Fig

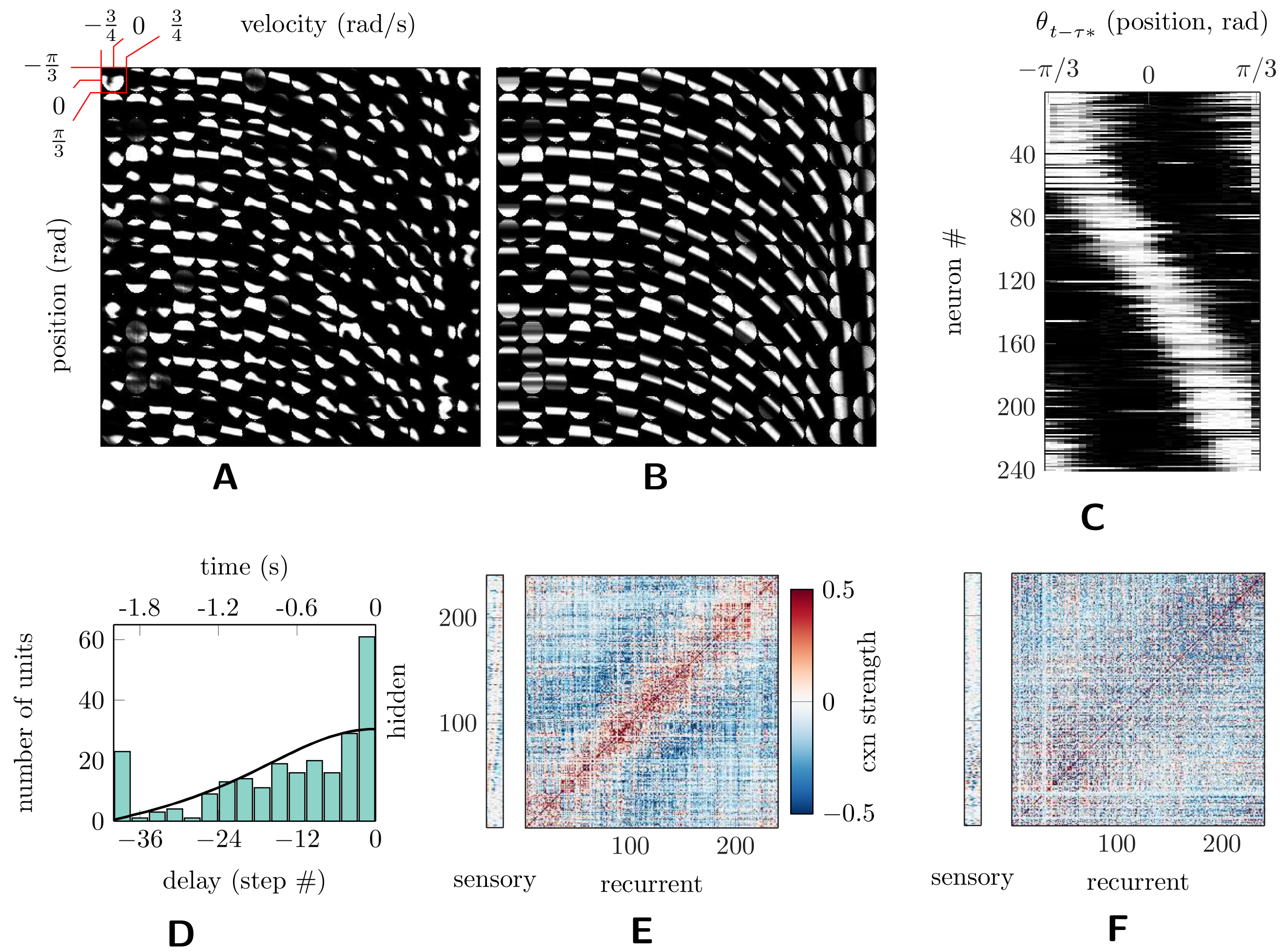

Fig S1. Receptive-field and weight-matrix analyses for the model of the section "Uncontrolled dynamical system" in the Results. The interpretation of the figures is identical to that in the corresponding figures for the "no-spring" model, Figs. 5, 7, and 8. The results are qualitatively similar, but the spring force in the underlying dynamics induces slightly more complicated receptive fields (RFs). The two major consequences are (1) under-representation in the corners of the receptive fields (RFs), and (2) a set of about 50 "anomalous" units, concentrated in the first two and last two columns of (A), that are not well explained by angle tuning at temporal delays (lags). (A) The RFs are sorted by preferred lag. In contrast to Fig. 5A, they are not "stripes," but segments of a disk. This is due to the spring force, which makes high speeds (left and right extremes of each sub-square) unlikely to co-occur with positions far from zero (top and bottom extremes of each sub-square). As a result, there are few data in these regions. Furthermore, anomalous units in the first two and last two columns do not follow this pattern at all. (B) The "idealized" RFs, which were constructed by assuming that the position tuning at the preferred lag, (C), completely explains the (non-lagged) position-velocity tuning shown in (A). Since these idealized RFs were evaluated, like the true RFs, only at trajectories from the generative model (in which there are essentially no samples at high-speed and highly nonzero positions), the disk again appears. The anomalous units are those sub-squares in (B) that do not resemble their counterparts in (A) (D) As in Fig. 5D, the distribution (across hidden units) of preferred lags matches well the autocorrelation of the angle being tracked—except for the bins at the extreme lags (-40, 0), which contain the anomalous units as well. (Here the autocorrelations are also longer than in the no-spring model, in part because of the spring force, and in part because the transition noise on the velocity was also larger in that model.) (E) The synaptic connections (weight matrix), where both the hidden units (ordinate) and the recurrent units (abscissa) have been sorted by the preferred angle of the hidden units. (F) The synaptic connections (weight matrix), where both the hidden units and the recurrent units have been sorted by the preferred lag of the hidden units. The connectivity resembles that in Fig. 8A,B , but the presence of anomalous units reduces the connectivity among neighbors at the lower-left and upper-right corners of the plot.
