# Supplementary material for: Learning to Estimate Dynamical State with Probabilistic Population Codes
Source: PLoS Comput Biol. 2015 Nov 5;11(11):e1004554. doi: 10.1371/journal.pcbi.1004554 (PMC4634970; doi:10.1371/journal.pcbi.1004554)
Supplement: S2 Fig — (PDF) [file pcbi.1004554.s005.pdf]

S2 Fig

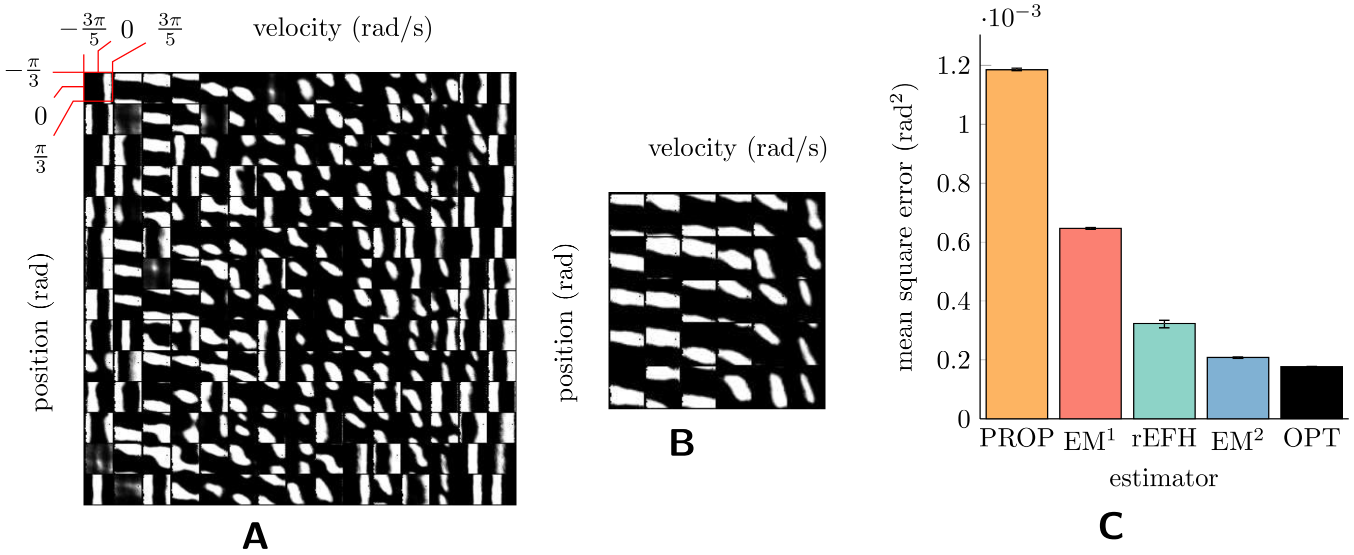

Fig S2. Receptive fields and performance for a model with velocity inputs. (A,B) Receptive fields. Within each square, position increases from top to bottom, and velocity increases from left to right. Units have again by sorted (approximately) by slope; cf. Figs. 5 and S1. The training data have the same dynamics as those for Fig. 5, but the observation model is different: both position and velocity are reported by PPCs. (A) All RFs. Unlike the RFs investigated above, many units in this rEFH show pure velocity tuning (vertical stripes). (B) A subset of the RFs in (A) that show tuning to lagged position, as in the previous models. (C) Performance of rEFHs and benchmark models on this generative model. As in Fig. 1E, the rEFH performs nearly as well as the second-order EM-based model (which is itself close to optimal), and much better than its first-order counterpart.
