# Supplementary material for: Learning to Estimate Dynamical State with Probabilistic Population Codes
Source: PLoS Comput Biol. 2015 Nov 5;11(11):e1004554. doi: 10.1371/journal.pcbi.1004554 (PMC4634970; doi:10.1371/journal.pcbi.1004554)
Supplement: S4 Text — (PDF) [file pcbi.1004554.s006.pdf]

S4 Text: Is the hidden layer a linear PPC?

Probabilistic population codes (PPCs) interpret the activities of a population of neurons, $\mathbf{r}$, as encoding a probability distribution over (as opposed to merely a point estimate of) the variable of interest in the world—what we call "the stimulus." In our case, this stimulus is the angle formed by the shoulder, θ. Thus, in a PPC, the meaning of neural activity across a population is given by a conditional distribution, $\Pr(\theta|\mathbf{r})$. In the rEFH, the "sensory" population is a PPC by construction. But the hidden layer must become one as well, since the optimal solution to the state estimation problem, which the network learns to solve, requires keeping track of precisely this distribution at each time step: The mean of this posterior distribution is the optimal estimate of joint angle, and its variance determines its reliability relative to the incoming sensory information, and therefore how these two sources of information about joint angle should be combined.

S4.1 Linear PPCs

Ma, Beck and colleagues [2, 7] have proposed that PPCs take a particular form. In particular, they argue that the *likelihood* function, $\Pr(\mathbf{r}|\theta)$, which characterizes the population's noisy response to a stimulus, (1) belong to an exponential family of probability distributions; (2) have linear sufficient statistics; and (3) encode stimulus reliability with a global gain, g, in such a way that, under the posterior distribution, stimulus location and reliability are independent: $\Pr(\theta|\mathbf{r}, g) = \Pr(\theta|\mathbf{r})$. (Thus, downstream neurons—or experimenters—need not know the gain to decode the stimulus itself from the population firing rates $\mathbf{r}$.) These are known as linear PPCs.

These constraints on the likelihood enforce constraints on the posterior, $\Pr(\theta|\mathbf{r})$. To begin with, only one of the summands in the log-likelihood involves both θ and $\mathbf{r}$:

$$\boldsymbol{\eta}(\theta, g)^{\mathrm{T}}\mathbf{t}_\theta(\mathbf{r}) = \boldsymbol{\eta}_\theta(\theta)^{\mathrm{T}}\mathbf{r} + \boldsymbol{\eta}_g(g)^{\mathrm{T}}\mathbf{r},$$

where $\boldsymbol{\eta}(\theta, g)$ is the vector of natural parameters of the likelihood, and $\mathbf{t}_\theta(\mathbf{r})$ is the vector of sufficient statistics. The right-hand side follows from conditions (2) and (3) above [2]. (*Linear* sufficient statistics imply that one may write simply $\mathbf{t}_\theta(\mathbf{r}) = \mathbf{r}$ because any matrix can be absorbed into the natural parameters.) Now, our posteriors are, by design, Gaussian, and therefore also in an exponential family, and thus can also be characterized in terms of natural parameters and sufficient statistics. Since the transformation from likelihood to posterior (Bayes's theorem) cannot introduce another term in which θ and $\mathbf{r}$ interact, these parameters can be deduced from the above equation; the roles are merely reversed:

$$\begin{aligned} \boldsymbol{\eta}_\mathbf{r}(\mathbf{r})^{\mathrm{T}}\mathbf{t}_\mathbf{r}(\theta) &= \boldsymbol{\eta}_\theta(\theta)^{\mathrm{T}}\mathbf{r} \\ \Longrightarrow \boldsymbol{\eta}_\mathbf{r}(\mathbf{r}) &= P\mathbf{r}, \end{aligned}$$

for some matrix P.

Therefore, if a population forms a linear PPC, the *natural parameters of the posterior distribution* should be recoverable with a linear decoder. The natural parameters of a (univariate) Gaussian—our posterior—are the mean-to-variance ratio ($\eta_{t,1}$), and the (scaled) inverse variance ($\eta_{t,2}$). Now, we have constructed the input "sensory" population—the likelihood $p(\mathbf{r}_t^\theta|\theta_t)$—to be a linear PPC, so the natural parameters of $p(\theta_t|\mathbf{r}_t^\theta)$ are indeed recoverable with a matrix from $\mathbf{r}_t^\theta$. What about the hidden layer?

S4.2 Decoding the natural parameters from the hidden layer

The likelihood $q(\mathbf{z}_t|\theta_t)$ is a distribution over binary vectors, and therefore expressible as a multivariate Bernoulli distribution; so it is in an exponential family, and satisfies condition (1). Now, the rEFH's performance is close to optimal, which means (as noted above) that the hidden units are able to read the full posterior distribution (at the previous time step) from the recurrent units—without access to the gain. Hence, $q(\theta_t|\mathbf{z}_t, g_t) \approx q(\theta_t|\mathbf{z}_t)$, which is condition (3). Finally, however, the likelihood is a *multivariate* Bernoulli distribution, not necessarily conditionally independent (given the stimulus), so the sufficient statistics are not necessarily linear (there may be terms of the form ${z_t}^i{z_t}^j$, etc.). Therefore it is necessary to check if indeed a linear decoder can recover from $\mathbf{z}_t$ the posterior natural parameters, $\mathrm{Var}_q[\Theta_t|\mathbf{z}_t]^{-1}\mathbb{E}_q[\Theta_t|\mathbf{z}_t] =: \eta_{t,1}$, and $-\frac{1}{2}\mathrm{Var}_q[\Theta_t|\mathbf{z}_t]^{-1} =: \eta_{t,2}$.

This question can be answered with linear regression: one simply finds the matrix P that minimizes squared error between $P\mathbf{z}_t$ and the natural parameters at each moment in time. But what do we use as the targets for

the regression? The posterior mean, $\mathbb{E}_q[\Theta_t|\mathbf{z}_t]$, can be estimated with the nonlinear decoder of the hidden units used throughout this paper (see **Testing** in the **Methods** of the main text); but the posterior variance is not so straightforward.

The posterior variance from OPT is truly optimal, but it does not make a good target for the regression: small discrepancies between dynamical models underlying OPT and the rEFH (or EM^2, e.g.) accumulate over the course of each trajectory, leading to significantly larger variances for the latter by late in the trial. (This is of course reflected in the differences in mean square error between these models.) Instead of the optimal posterior variance, then, we use the posterior variance of the Kalman filter EM^2, since the rEFH and EM^2 appear to have learned similar models. We then compute $\mathrm{Var}_{\mathrm{EM}^2}[\Theta_t|\mathbf{r}_1^\theta, \ldots, \mathbf{r}_t^\theta]^{-1}\mathbb{E}_q[\Theta_t|\mathbf{z}_t]$ and $-\frac{1}{2}\mathrm{Var}_{\mathrm{EM}^2}[\Theta_t|\mathbf{r}_1^\theta, \ldots, \mathbf{r}_t^\theta]^{-1}$ for all time, and use these natural parameters as the targets for a linear regression that takes the hidden-unit activities, $\mathbf{z}_t$, as the inputs. To determine whether the hidden layer really does encode these parameters linearly—and therefore, whether it constitutes a linear PPC—we examine the regression fits on "held-out" data, Fig. S3.

For comparison, we begin with a regression model for the posterior mean itself—not one of the natural parameters. The linear decoding appears quite good (Fig. S3A), with a coefficient of determination of 0.997, and no large asymmetries of the residuals—although we note that it is nevertheless inferior to the nonlinear decoding of the posterior mean used throughout the paper ($\mathrm{R}^2 = 0.999$). Fig. S3B shows the regression fit for η_1, which also appears to be essentially linear, with a high R^2 of 0.992. Nevertheless, that linear decoding is more accurate for the posterior mean than for a posterior natural parameter already suggests that the hidden units do not constitute a linear PPC. Moreover, for η_2, the fit is much worse (Fig. S3C, $\mathrm{R}^2 = 0.415$). It is not, on the other hand, immediately obvious how accurately even an optimal (possibly nonlinear) decoder can reconstruct the inverse posterior variance. Therefore, to determine whether indeed the linear decoder has lost information, we repeated the regression for η_2 with nonlinear terms among the inputs; in particular, we included a random subset of 240 interaction terms of the form ${z_t}^i {z_t}^j$. The resulting decoder achieves superior prediction (Fig. S3D, $\mathrm{R}^2 = 0.544$). This increase in performance cannot be explained by overfitting because the regression was evaluated on a held-out set of trajectories. Since a nonlinear transformation of the hidden activities improves the mapping from hidden units to posterior natural parameters, the relationship is nonlinear, and condition (2) is not satisfied. We conclude that the hidden units are not a linear PPC.
