# Supplementary material for: Learning to Estimate Dynamical State with Probabilistic Population Codes
Source: PLoS Comput Biol. 2015 Nov 5;11(11):e1004554. doi: 10.1371/journal.pcbi.1004554 (PMC4634970; doi:10.1371/journal.pcbi.1004554)
Supplement: S3 Fig — (PDF) [file pcbi.1004554.s007.pdf]

S3 Fig

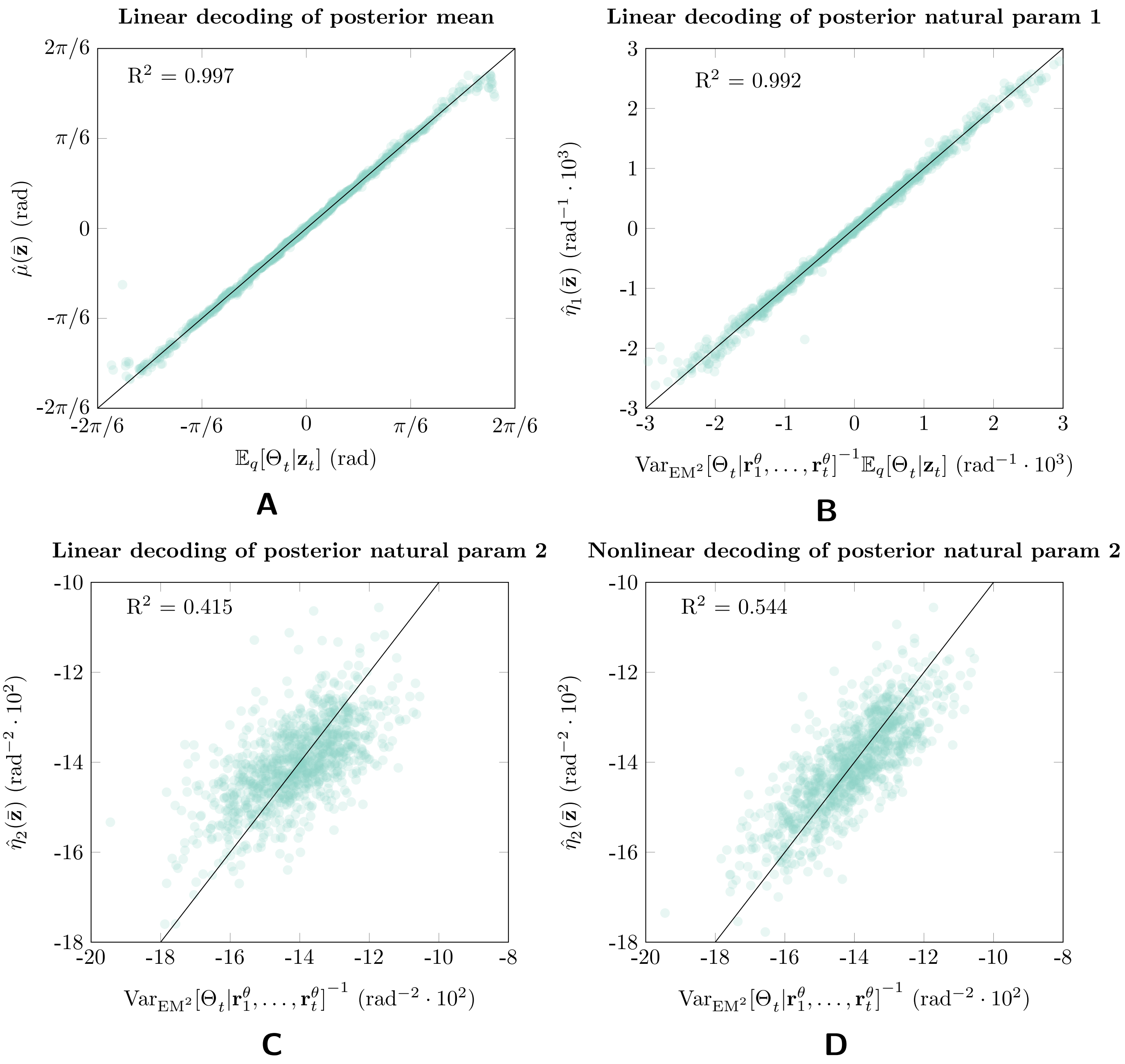

Fig S1. Is the hidden layer of the rEFH a linear PPC? If it is, the natural parameters of the posterior distribution should be linearly decodable from the activities of that layer. In these plots, the posterior variance was computed with the Kalman filter EM^2; the posterior mean was decoded from the hidden units as throughout the paper (see **Testing** in the **Methods** of the main text). Linearly decoded parameters (ordinates) are plotted against true parameters (abscissae), for all trajectories and all time. (Hence, each point in the plots corresponds to a single discrete time in one of the trajectories. All data are from testing, rather than training, trajectories.) Linear decoders were acquired by linear regression on training data. (A) Linear decoding of the posterior mean from the hidden units. (B) Linear decoding of η_1, the mean-to-variance ratio. (C) Linear decoding of η_2, the inverse variance of the posterior. (D) A nonlinear decoding of η_2 (see text for details). That the fit is superior to (C) suggests that the hidden layer is not a linear PPC.
