# Supplementary material for: Learning to Estimate Dynamical State with Probabilistic Population Codes
Source: PLoS Comput Biol. 2015 Nov 5;11(11):e1004554. doi: 10.1371/journal.pcbi.1004554 (PMC4634970; doi:10.1371/journal.pcbi.1004554)
Supplement: S1 Bibliography — (PDF) [file pcbi.1004554.s008.pdf]

S1 Bibliography

[1] Beck JM, Latham PE, Pouget A. Marginalization in Neural Circuits with Divisive Normalization - Supporting Information. Journal of Neuroscience. 2011;31(43).

[2] Ma WJ, Beck JM, Latham PE, Pouget A. Bayesian Inference with Probabilistic Population Codes. Nature Neuroscience. 2006;9:1423–1438.

[3] Makin JG, Fellows MR, Sabes PN. Learning Multisensory Integration and Coordinate Transformation via Density Estimation - Supporting Material. PLoS Computational Biology. 2013;9(4):1–9.

[4] Ghahramani Z, Hinton GE. Parameter Estimation for Linear Dynamical Systems. University of Toronto; 1996.

[5] Makin JG, Fellows MR, Sabes PN. Learning Multisensory Integration and Coordinate Transformation via Density Estimation. PLoS Computational Biology. 2013;9(4):1–17.

[6] Beck JM, Latham PE, Pouget A. Marginalization in Neural Circuits with Divisive Normalization. Journal of Neuroscience. 2011 oct;31(43):15310–9.
